# Ultra-thin metal-organic framework nanoribbons

**DOI:** 10.1093/nsr/nwz118

**Published:** 2019-08-13

**Authors:** Bingqing Wang, Meiting Zhao, Liuxiao Li, Ying Huang, Xiao Zhang, Chong Guo, Zhicheng Zhang, Hongfei Cheng, Wenxian Liu, Jing Shang, Jing Jin, Xiaoming Sun, Junfeng Liu, Hua Zhang

**Affiliations:** 1 State Key Laboratory of Chemical Resource Engineering, Beijing University of Chemical Technology, Beijing 100029, China; 2 Center for Programmable Materials, School of Materials Science and Engineering, Nanyang Technological University, Singapore 639798, Singapore; 3 Department of Chemistry, City University of Hong Kong, Hong Kong, China

**Keywords:** metal-organic frameworks, nanoribbons, metal-hydroxide nanostructures, structure engineering, DNA detection

## Abstract

Structure engineering of metal-organic frameworks (MOFs) at the nanometer scale is attracting increasing interest due to their unique properties and new functions that normally cannot be achieved in bulk MOF crystals. Here, we report the preparation of ultra-thin MOF nanoribbons (NRBs) by using metal-hydroxide nanostructures as the precursors. Importantly, this general method can be used to synthesize various kinds of ultra-thin MOF NRBs, such as MBDC (M = Co, Ni; BDC = 1,4-benzenedicarboxylate), NiCoBDC, CoTCPP (TCPP = tetrakis(4-carboxyphenyl)porphyrin) and MIL-53(Al) NRBs. As a proof-of-concept application, the as-prepared ultra-thin CoBDC NRBs have been successfully used as a fluorescent sensing platform for DNA detection, which exhibited excellent sensitivity and selectivity. The present strategy might open an avenue to prepare MOF nanomaterials with new structures and unique properties for various promising applications.

## INTRODUCTION

Metal-organic frameworks (MOFs) are an intriguing class of functional materials, possessing many noticeable features, such as a large surface area, highly ordered pores, tunable structure and unique function, making them promising for gas separation [1,2], gas storage [3], sensing [4], catalysis [5–10] and so on. However, the normal bulk MOF crystals cannot meet all the requirements of some specific applications. Therefore, the structure engineering of MOFs at the nanometer scale has gained increasing interest due to their unique size- and shape-dependent properties, which are essential to customize MOFs for specific applications [11–16]. For instance, the crystal downsizing of Cu_2_(BDC)_2_(BPY) (DBC = 1,4-benzenedicarboxylate, BPY = 4,4′-bipyridine) leads to the formation of MOF nanosheets with shape-memory effect, which has not been observed in the bulk MOF crystal [17]. In addition, ultra-thin MOF nanosheets have been demonstrated to exhibit improved properties in gas separation [18,19], sensing [20,21] and catalysis [22–24], as compared to their bulk counterparts. Very recently, MOF crystals with novel structures, such as ordered macro-microporous and multi-shelled hollow nanostructures, have been synthesized, which showed significantly enhanced catalytic activities [14,25]. Therefore, structure engineering of MOFs at the nanometer scale provides a new way to tailor the properties of MOFs for various promising applications.

Ultra-thin nanoribbons (NRBs), as a class of quasi-1D nanostructures, have attracted both fundamental and technological interests over the past decades due to their high surface-to-volume ratio, highly active surface and high concentration of selectively exposed crystal facet. These features enable them to exhibit unique electronic structures [26,27], mechanical properties [28] and excellent catalytic efficiency [28,29]. Until now, various kinds of inorganic NRBs have been prepared, such as metal oxides [27], graphene [30] and noble metals [31]. However, the preparation of ultra-thin MOF NRBs still remains a great challenge due to the complicated nucleation and growth processes of MOFs [32]. The preparation of MOF NRBs requires the anisotropic growth of MOFs, i.e. the growth of MOFs preferentially follows a specific direction but no growth or very slow growth in the other directions [33]. This is very difficult to achieve due to the 3D structure of MOF crystals [34].

**Scheme 1.**
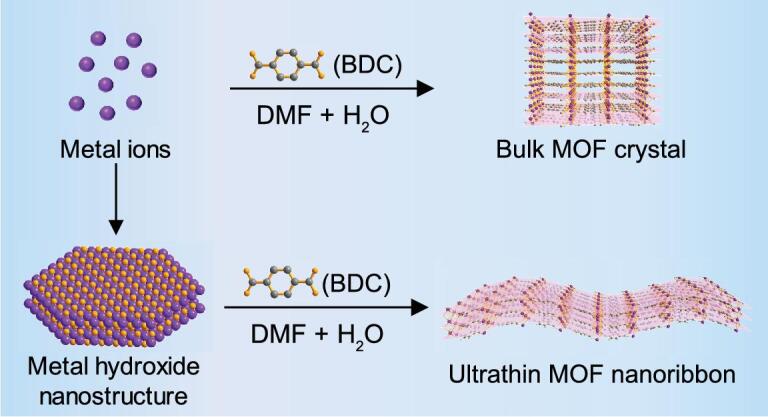
Comparision of the traditional approach to bulk MOF crystal (top) and metal-hydroxide nanostructure precursor approach to ultra-thin MOF nanoribbon (bottom).

Herein, we report a general method to prepare ultra-thin MOF NRBs by using the metal-hydroxide nanostructures as precursors. The obtained MOFs showed quasi-1D ribbon-like morphology with thickness of less than 10 nm. Importantly, the proposed method is simple, efficient and versatile, and could be used for the preparation of a series of ultra-thin MOF NRBs. As a proof-of-concept application, the as-prepared ultra-thin MOF NRBs exhibited excellent sensitivity and selectivity in the DNA detection.

## RESULTS AND DISCUSSION

**Figure 1.**
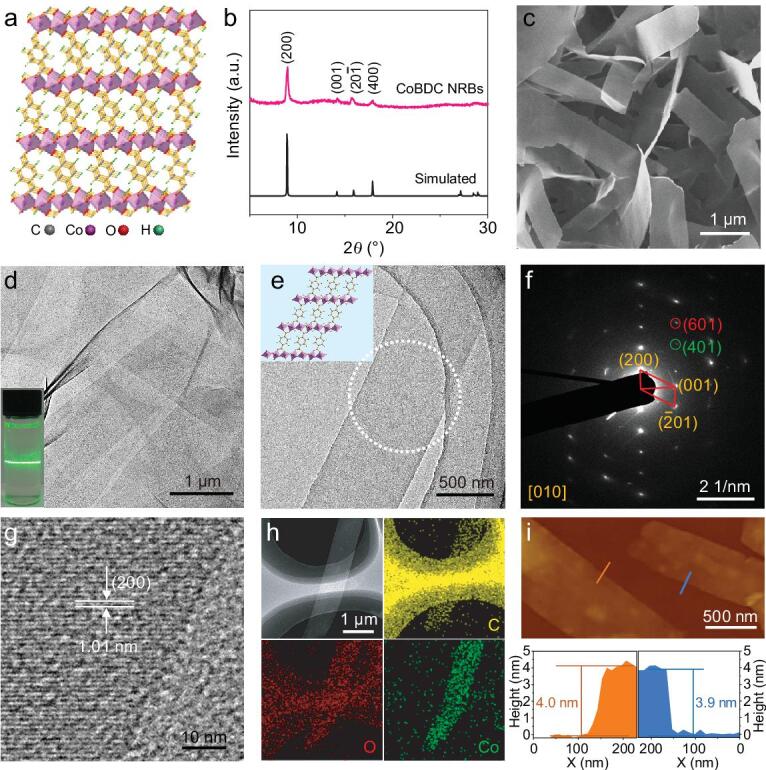
(a) Crystal structure of CoBDC. (b) XRD pattern, (c) SEM and (d) TEM images of the as-prepared CoBDC NRBs. Inset in (d): the Tyndall effect of colloidal CoBDC NRBs in ethanol. (e) TEM image of an individual CoBDC NRB and (f) the corresponding SAED pattern of the dotted circular area in (e). Inset in (e): the corresponding crystal structure of CoBDC NRB in the (010) plane. (g) HRTEM image of a CoBDC NRB taken at edge area of a CoBDC NRB. (h) Dark-field STEM image and the corresponding EDX element mappings of a CoBDC NRB. (i) AFM images of CoBDC NRBs and the corresponding height profiles measured along the orange and blue lines in (i), respectively.

The synthesis of ultra-thin MOF NRBs is based on the controlled transformation of metal-hydroxide nanostructures via a dissolution–reprecipitation process [35–37] (Scheme 1; see the experimental section in Supporting Information (SI) for details). To illustrate our synthesis approach, the Co_2_(OH)_2_(BDC), denoted as CoBDC, is taken as an example. Note that the conventional solvothermal synthesis, which uses cobalt salt as the metal source, mainly produces quadrilateral CoBDC crystals with edge lengths of 21.4 ± 6.0 and 13.0 ± 3.2 μm, respectively (Supplementary Fig. S1). However, when the cobalt-hydroxide hexagonal nanoplates [38] (Supplementary Fig. S2) were used as the metal source, ultra-thin CoBDC NRBs were successfully obtained after reacting with BDC at 50°C for 12 h. The morphology, structure and composition of the as-obtained ultra-thin CoBDC NRBs were characterized by various techniques (Fig. 1). Fig. 1a schematically illustrates the crystal structure of CoBDC MOF, which is assembled by cobalt species and BDC ligands [22]. The powder X-ray diffraction (XRD) pattern of the obtained product matches well with the simulated crystal structure, indicating the successful synthesis of CoBDC MOF (Fig. 1b). The scanning electron microscopy (SEM) image clearly shows the strip-like morphology of CoBDC NRBs (Fig. 1c) with a length of 3.8 ± 0.8 μm and width of 610.6 ± 93.1 nm (Supplementary Fig. S3). The transmission electron microscopy (TEM) image shows that the CoBDC NRBs are very thin, as confirmed by their low contrast and easy folding at the edge (Fig. 1d). The suspension of CoBDC NRBs in ethanol showed the typical Tyndall effect when a green laser passed through it, confirming their colloidal nature (inset in Fig. 1d) [20]. The selected area electron diffraction (SAED) pattern collected from an individual CoBDC NRB shows diffraction spots, which can be attributed to the (200), (001) and ($\overline{2}$01) planes (Fig. 1e and f) and is consistent with the simulated ones along the [010] axis (Supplementary Fig. S4a and b), confirming their single-crystalline structure. The high-resolution TEM (HRTEM) image taken from the edge of an individual CoBDC NRB shows lattice fringes with the interplanar distance of 1.01 nm, which corresponds to the (200) plane of CoBDC MOF (Fig. 1g), further confirming the crystal structure of CoBDC NRBs. A typical dark-field scanning TEM (STEM) image and corresponding STEM-energy-dispersive X-ray spectroscopy (EDS) element mappings (Fig. 1h) indicate that the obtained NRBs are composed of C, O and Co elements, which are uniformly distributed in the CoBDC NRB. The thickness of the CoBDC NRBs is about 4.1 ± 1.1 nm (Fig. 1i and Supplementary Fig. S5), as determined by atomic force microscopy (AFM), corresponding to about 13 packing layers of CoBDC NRB along [010] (Supplementary Fig. S4c and d). The specific surface area of CoBDC NRBs measured using the Brunauer-Emmett-Teller is 65.2 m^2^ g^−1^, which is about three times that of the bulk CoBDC crystals (23.4 m^2^ g^−1^) (Supplementary Fig. S6a). Thermal stability of CoBDC NRBs was investigated using thermogravimetric analysis (TGA). As shown in Supplementary Fig. S6b, the CoBDC NRBs are stable up to 400°C. Note that, when the temperature increased to 400°C, a higher weight loss of the CoBDC NRBs than that of the bulk CoBDC was observed, corresponding to the loss of more adsorbed solvent on CoBDC NRBs [23].

**Figure 2.**
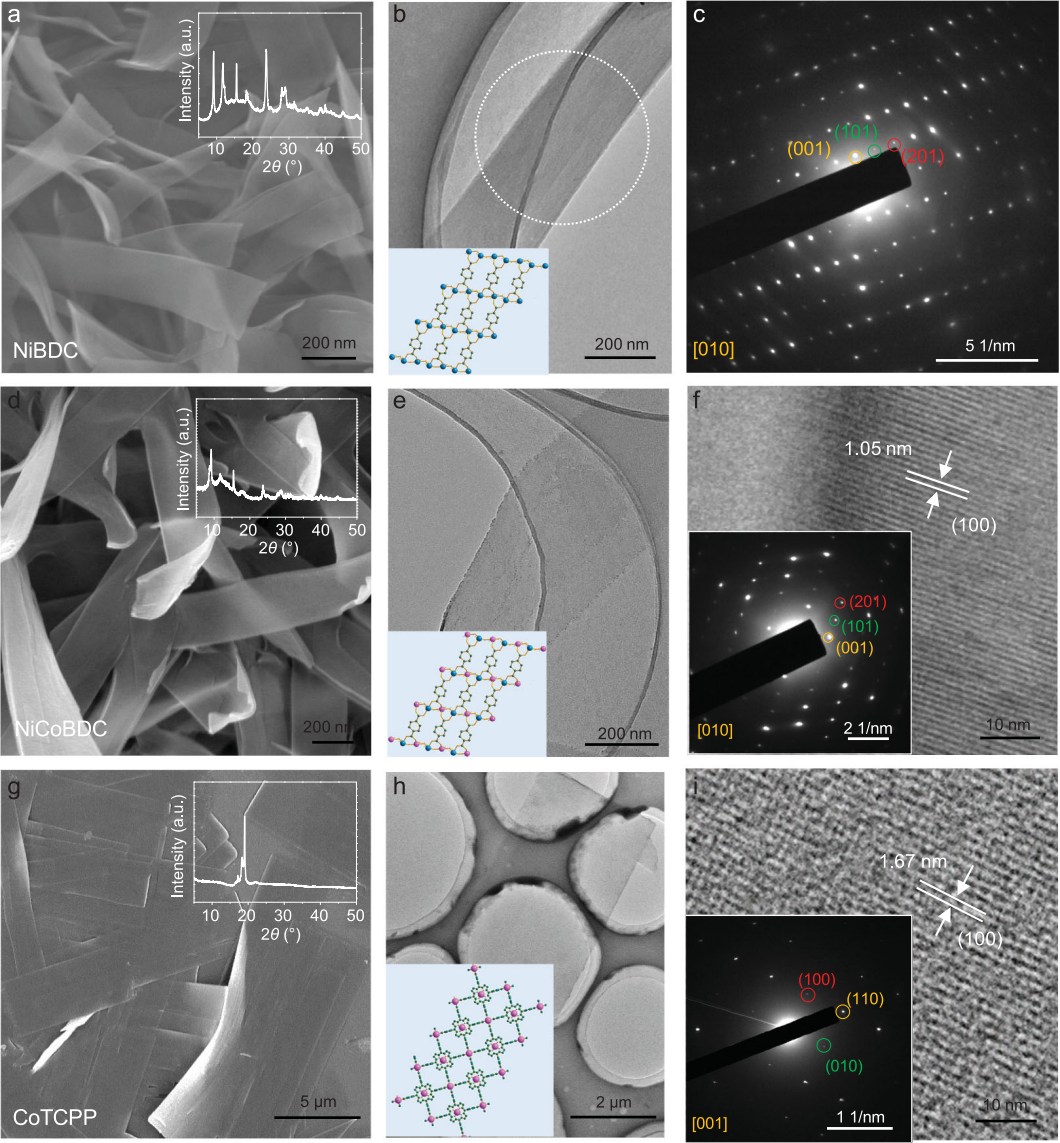
Synthesis of various kinds of ultra-thin MOF NRBs. (a) SEM image of NiBDC NRBs. Inset: XRD pattern of NiBDC NRBs. (b) TEM image and (c) the corresponding SAED pattern of the selected region (white dotted circle) of a NiBDC NRB. Inset in (b): the corresponding crystal structure of NiBDC NRB in the (010) plane. (d) SEM image of NiCoBDC NRBs. Inset: XRD pattern of NiCoBDC NRBs. (e) TEM and (f) HRTEM images of a NiCoBDC NRB. Inset in (e): the corresponding crystal structure of NiCoBDC NRB in the (010) plane. Inset in (f): the corresponding SAED pattern. (g) SEM image of CoTCPP NRBs. Inset: XRD pattern of CoTCPP NRBs. (h) TEM and (i) HRTEM images of a CoTCPP NRB. Insets in (h): the corresponding crystal structure of CoTCPP NRB in the (001) plane. Insets in (i): the corresponding SAED pattern.

In order to study the formation of CoBDC NRBs, the morphology and composition evolution from Co(OH)_2_ nanoplates to CoBDC NRBs were studied (Supplementary Fig. S7). The edges of Co(OH)_2_ nanoplates (Supplementary Fig. S7a) first became ambiguous after reaction of 20 min (Supplementary Fig. S7b) and the growth of NRBs along the Co(OH)_2_ nanoplates was observed after reaction of 30 min (Supplementary Fig. S7c). At prolonged reaction time, e.g. 50 min, the Co(OH)_2_ nanoplates vanished and only ultra-thin CoBDC NRBs were observed (Supplementary Fig. S7d). This conversion process was also confirmed by XRD and Fourier Transform Infrared (FTIR) measurements (Supplementary Fig. S7e and f). It is worth mentioning that the concentration of Co^2+^ during the conversion remained at a relatively low value (less than 0.2 mM) (red curve in Supplementary Fig. S8a), indicating that the dissolved Co^2+^ from the Co(OH)_2_ nanoplates can be immediately captured by the BDC ligands to form the CoBDC NRBs. Therefore, the growth behavior of CoBDC was controlled by the dissolution rate of the Co(OH)_2_ nanoplates, which determined the formation of ultra-thin CoBDC NRBs [39]. In addition, the morphology and crystallinity effects of the Co(OH)_2_ precursors on the synthesis of CoBDC NRBs were investigated. When irregular nanoparticles or low crystalline nanosheets of Co (OH)_2_ were used as the precursors, CoBDC NRBs could also be obtained (Supplementary Figs S9 and S10).

Importantly, this approach is general and can be used to synthesize other MOF NRBs from their corresponding metal-hydroxide nanostructures. For example, the Ni_3_(OH)_2_(BDC)_2_(H_2_O)_4_ (denoted as NiBDC) NRBs were synthesized using nickel hydroxide nanoplates [40] (Supplementary Fig. S11a and b) as precursors and BDC as the ligands (see the experimental section in SI for details). The SEM image in Fig. 2a shows the flexible structure of the obtained NiBDC NRBs. The low contrast of the TEM image of a representative NRB indicates its ultra-thin thickness (Fig. 2b). The corresponding SAED pattern taken on the NRB shows its single-crystalline nature (Fig. 2c). The XRD peaks of the ultra-thin NRBs (inset in Fig. 2a) are consistent with the reported NiBDC (CCDC No. 638866, Cambridge Crystallographic Data Centre) [41], indicating the successful preparation of Ni-based MOF NRBs. The EDS mappings confirmed the homogeneous distribution of C, O and Ni elements in the NiBDC NRBs (Supplementary Fig. S12). In addition, when the Ni(OH)_2_ nanoparticles (Supplementary Fig. S13a and b) were used as the precursors, the NiBDC NRBs could also be obtained (Supplementary Fig. S13c and d). Besides the Co and Ni-based MOF NRBs, the Al-based MIL-53(Al) NRBs were also successfully synthesized using AlO(OH) nanosheets as the precursors (Supplementary Figs S14 and S15).

Moreover, this facile method can also be used to prepare ultra-thin multicomponent MOF NRBs by simply using multi-metal-hydroxide nanosheets as precursors. For example, bimetallic NiCoBDC NRBs were prepared using the NiCo-hydroxide nanosheets (Supplementary Fig. S11c and d), which were synthesized using a previously reported method [42] with slight modification, as precursors to react with BDC ligands (see the experimental section in SI for details). The SEM image (Fig. 2d) shows the morphology of obtained NRBs with the curled edge, indicating their ultra-thin nature, which is consistent with the TEM result (Fig. 2e). The HRTEM image (Fig. 2f) shows lattice fringes of the (100) planes with a spacing of 1.05 nm and the SAED pattern (inset in Fig. 2f) shows the spots of the (001), (101) and (201) planes, indicating its single-crystalline nature. The XRD measurement demonstrates that the NiCoBDC NRBs are in a single phase that is isostructural to the NiBDC NRBs (inset in Fig. 2d). The EDS and EDS elements mapping analysis confirm that the obtained NRBs are composed of C, O, Co and Ni elements, which homogeneously distribute in the NRB (Supplementary Figs S16 and S17), implying the successful synthesis of bimetallic NiCoBDC NRBs.

**Figure 3.**
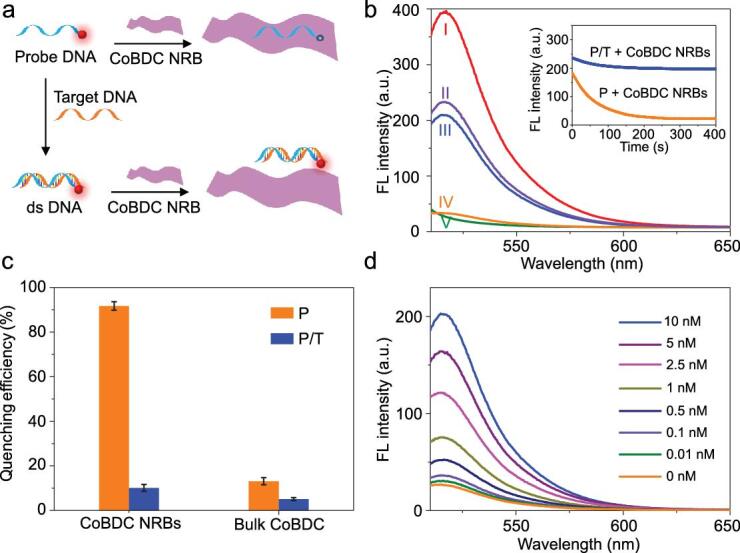
Detection of DNA with CoBDC NRBs. (a) Schematic illustration of a CoBDC NRB-based fluorescence sensor for detection of DNA. (b) Fluorescence spectra under different experimental conditions: (I) P; (II) P/T; (III) P/T + CoBDC NRBs; (IV) P + CoBDC NRBs; and (V) CoBDC NRBs. The concentrations of P, T and CoBDC NRBs in the final solution are 10 nM, 10 nM and 30 μg mL^−1^, respectively. Inset: kinetic study on the fluorescence change of P and P/T in the presence of CoBDC NRBs. Excitation and emission wavelengths are 494 and 516 nm, respectively. (c) Fluorescence quenching efficiency of CoBDC NRBs and bulk CoBDC. The concentrations of P, T, CoBDC NRBs and bulk CoBDC in the final solution are 10 nM, 10 nM, 30 μg mL^−1^ and 30 μg mL^−1^, respectively. (d) Fluorescence spectra of P (10 nM) in the presence of different concentrations of T with addition of CoBDC NRBs. The final concentration of CoBDC NRBs is 30 μg mL^−1^.

Impressively, our proposed method can also be used to prepare MOF NRBs with other organic ligands. When TCPP (TCPP = tetrakis(4-carboxyphenyl)porphyrin) was used as ligand instead of BDC, CoTCPP NRBs were successfully synthesized using the same strategy (see the experimental section in SI for details). The ribbon-like structure of CoTCPP was confirmed by SEM and TEM images (Fig. 2g and h). Lattice fringes with an interplanar distance of 1.67 nm were observed by HRTEM, corresponding to the (100) planes of CoTCPP (Fig. 2i). Moreover, the SAED pattern was acquired along the [001] axis and showed diffraction spots corresponding to the (hk0) planes of the CoTCPP NRB (inset in Fig. 2i). The XRD pattern of CoTCPP NRBs only shows a strong reflection that belongs to the (004) plane (inset in Fig. 2g), indicating the preferential orientation of the ultra-thin NRBs on the glass substrate [20]. The EDS mappings confirmed the homogeneous distribution of C, O, N and Co elements (Supplementary Fig. S18). AFM results confirmed that the thicknesses of the aforementioned NiBDC, NiCoBDC and CoTCPP NRBs are less than 10 nm (Supplementary Fig. S19). All these results prove that the metal-hydroxide nanostructure precursor approach is simple and versatile to synthesize a series of ultra-thin quasi-1D MOF NRBs.

Recently, 2D nanomaterials, such as graphene [43,44], transition metal dichalcogenides (TMDs) [45–47], covalent organic frameworks (COFs) [48] and MOFs [20], have been successfully used for DNA detection. As a proof-of-concept application, CoBDC NRBs were used as the sensing platform for DNA detection. As schematically shown in Fig. 3a, a single-stranded DNA (ssDNA) probe is labeled with a fluorescent dye, showing strong fluorescence emission in the absence of CoBDC NRBs. After the ssDNA probe is mixed with the CoBDC NRBs, it is adsorbed on the surface of CoBDC NRBs due to the π − π stacking [48] and hydrogen bonding [49], resulting in the fluorescence quenching of the dye. However, when the complementary target DNA is added, it can hybridize with the ssDNA probe to form the double-stranded DNA (dsDNA). The resulted dsDNA has a weak interaction with the CoBDC NRBs, which makes the dye-labeled probe away from the surface of the CoBDC NRB and results in a low fluorescence quenching of the dye in the presence of CoBDC NRBs [20,47]. Therefore, we expect that the fluorescence intensity of the probe can provide a quantitative detection of the target DNA.

In our experiment, the used DNA sequences are listed in Supplementary Table S1. As shown in Fig. 3b, the 6-carboxyfluorescein (FAM)-labeled ssDNA probe (denoted as P) shows strong emission at the wavelength of 516 nm (curve I). After addition of CoBDC NRBs, the fluorescence intensity of P is quenched by about 91% within 5 min (curve IV and orange curve in the inset in Fig. 3b), suggesting the strong fluorescence quenching ability of CoBDC NRBs. In contrast, the fluorescence intensity can be greatly retained after the hybridization of P with the target complementary DNA (H1N1, denoted as T) even in the presence of CoBDC NRBs (Fig. 3b, curve III and blue curve in inset). Note that the decreased fluorescence intensity of the P/T duplex (Fig. 3b, curve II) than the P at 516 nm in the absence of CoBDC NRBs (Fig. 3b, curve I) can be attributed to the different effect of P and T DNA structures on the fluorescence properties of labeled dyes [50]. Compared to the bulk CoBDC MOFs, the CoBDC NRBs exhibit an excellent fluorescence quenching ability (Fig. 3c), which could be attributed to the increased surface area of CoBDC NRBs [20].

In order to confirm the sensitivity of the CoBDC NRBs in DNA detection, different concentrations of T were hybridized with P, followed by the addition of CoBDC NRBs. As shown in Fig. 3d and Supplementary Fig. S20, the fluorescence intensity was enhanced obviously alongside the increase in the concentration of T and exhibited a linear relationship over the range from 0 to 2.5 nM with the detection limit of 20 pM, which is much lower than that of the previously reported MOF-based DNA sensors and also comparable with other 2D nanomaterials-based sensors (Supplementary Table S2). Furthermore, the selectivity of this CoBDC NRB-based sensor was evaluated using the complementary target DNA (T), single-base mismatch DNA (SM) and random DNA (R). The obtained high fluorescent signal of T compared with the SM or R indicated that the CoBDC NRB-based sensor exhibited excellent selectivity for the target DNA (Supplementary Fig. S21). Importantly, no obvious change was observed in the structure of CoBDC NRBs and no ligand leaching was detected in the supernatant fluid after the DNA detection, indicating the excellent stability of CoBDC NRBs (Supplementary Fig. S22).

## CONCLUSIONS

In summary, we report an efficient and versatile method to synthesize a series of ultra-thin MOF NRBs, including CoBDC, NiBDC, NiCoBDC, CoTCPP and MIL-53(Al) NRBs, using the related metal-hydroxide nanostructures as precursors. As a proof-of-concept application, CoBDC NRBs are successfully used for DNA detection, which exhibited excellent sensitivity and selectivity. More importantly, our strategy using metal-hydroxide nanostructures as precursors can control the growth of MOF crystals by releasing metal ions from the metal hydroxides, which plays a key role in the synthesis of MOF NRBs. This strategy may open up a new way for engineering the structures, facets, sizes, dimensions, shapes, architectures, lattice strains and compositions of MOFs at the nanometer scale, which could exhibit unique physicochemical properties and various promising applications.

## Supplementary Material

nwz118_Supplemental_File
