# Supplementary material for: Ultra-thin metal-organic framework nanoribbons
Source: Natl Sci Rev. 2019 Aug 13;7(1):46–52. doi: 10.1093/nsr/nwz118 (PMC8288949; doi:10.1093/nsr/nwz118)
Supplement: nwz118_Supplemental_File [file nwz118_supplemental_file.docx]

**Supporting Information**

**Ultrathin Metal-Organic Framework Nanoribbons**

*Bingqing Wang^1,2,^*^†^*, Meiting Zhao^2,^*^†^*, Liuxiao Li^2,^*^†^*, Ying Huang^2^, Xiao Zhang^2^, Chong Guo^1^, Zhicheng Zhang^2^, Hongfei Cheng^2^, Wenxian Liu^1^, Jing Shang^1^, Jing Jin^1^, Xiaoming Sun^1^, Junfeng Liu^1^* and Hua Zhang^2,3^**

^1^State Key Laboratory of Chemical Resource Engineering, Beijing University of Chemical Technology, Beijing 100029, P.R. China

^2^Center for Programmable Materials, School of Materials Science and Engineering, Nanyang Technological University, 50 Nanyang Avenue, Singapore 639798, Singapore

^3^Department of Chemistry, City University of Hong Kong, Kowloon, Hong Kong, China.

^†^These authors contributed equally to this work.

*Corresponding authors

E-mail: ljf@mail.buct.edu.cn

E-mail: [hzhang@ntu.edu.sg](mailto:hzhang@ntu.edu.sg); hua.zhang@cityu.edu.hk

**Experimental Section
Materials.** Cobalt chloride hexahydrate (CoCl_2_·6H_2_O, 98%), nickel acetate tetrahydrate (Ni(CH_3_COO)_2_·4H_2_O, 98%), nickel chloride hexahydrate (NiCl_2_·6H_2_O, 98%), N, N-dimethylformamide (DMF, 99.8%), 1,4-benzenedicarboxylate (BDC, 99%) were purchased from Sigma-Aldrich. Tetrakis(4-carboxyphenyl)porphyrin (TCPP, 97%) was purchased from Tokyo Chemical Industry Corporation Ltd. Hexamethylenetetramine (HMT) and NH_3_·H_2_O were purchased from China National Pharmaceutical Industry Corporation Ltd. All DNA strands were synthesized and purified by the Integrated DNA Technologies Pte Ltd (Singapore). The deionized water was obtained from the Milli-Q System. All the materials were used as received without further purification.

**Synthesis of hydroxide precursors.**

***Synthesis of Co(OH)_2_ nanoplates***[1]**.** In a typical procedure, CoCl_2_·6H_2_O (1.0 mmol) and HMT (12.0 mmol) were dissolved in a mixed solution of 180 mL deionized water and 20 mL ethanol. Then the solution was heated at 90 ℃ for 1 h under magnetic stirring and a pink-colored suspension was generated. The solid product of Co(OH)_2_ nanoplates were obtained by centrifuging at 8000 rpm and washing with deionized water for 3 times.

***Synthesis of Ni(OH)_2_ nanoplates***[2]**.** In a typical procedure, Ni(CH_3_COO)_2_·4H_2_O (1.40 mmol) was dissolved in a 15.0 mL deionized water, and then 0.5 mL of 6.0 M NH_3_·H_2_O was added dropwise during magnetic stirring. Then the solution was sealed in a 30 mL Teflon autoclave and heated at 200 °C for 5 h. After hydrothermal reaction, a green suspension was generated. The solid product of Ni(OH)_2_ nanoplates were obtained by centrifuging at 8000 rpm and washing with deionized water for 3 times.

***Synthesis of*** ***NiCo hydroxide nanosheets*.** The synthesis of NiCo hydroxide nanosheets was based on a previous report [3] with slight modification. In a typical procedure, NiCl_2_·6H_2_O (1.25 mmol) and CoCl_2_·6H_2_O (2.50 mmol) were firstly dissolved in 500 mL deionized water, and then 45.0 mmol of HMT was added. The solution was refluxed at 95 ℃ for 5 h under continuous magnetic stirring. The solid product of Ni_1/2_Co_2/3_(OH)_2_ nanosheets were obtained centrifuging at 8000 rpm and washing with deionized water for 3 times.

***Synthesis of AlO(OH) nanosheets*.** In a typical procedure, Aluminium isopropoxide (3.0 mmol) was dissolved in 5.4 mL deionized water under continuous magnetic stirring, and then the solution was heated at 70 ℃ for 1.5 h. The solid product of AlO(OH) nanosheets were obtained centrifuging at 8000 rpm and washing with deionized water for 3 times.

**Synthesis of MOF nanoribbons (NRBs).**

***Synthesis of CoBDC NRBs*.** The pre-prepared Co(OH)_2_ nanoplates (0.021 mmol) and BDC (0.024 mmol) were added in a mixed solution of 3 mL DMF and 1 mL deionized water in a 20 mL capped vial under stirring. After that, the vial was heated to 50 ℃ and then kept for 12 h. The resulting pink CoBDC NRBs were washed twice with DMF and ethanol, respectively. Finally, the obtained CoBDC NRBs were redispersed in ethanol.

***Synthesis of NiBDC NRBs*.** The pre-prepared Ni(OH)_2_ nanoplates (0.021 mmol) and BDC (0.024 mmol) were added in a mixed solution of 3 mL DMF and 1 mL deionized water in a 20 mL capped vial under stirring. After that, the vial was heated to 60 ℃ and then kept for 12 h. The resulting light green NiBDC NRBs were washed twice with DMF and ethanol, respectively. Finally, the obtained NiBDC NRBs were redispersed in ethanol.

***Synthesis of NiCoBDC NRBs*.** The pre-prepared NiCo hydroxide nanosheets (0.021 mmol) and BDC (0.024 mmol) were added in a mixed solution of 3 mL DMF and 1 mL deionized water in a 20 mL capped vial under stirring. After that, the vial was heated to 50 ℃ and then kept for 12 h. The resulting gray-green NiCoBDC NRBs were washed twice with DMF and ethanol, respectively. Finally, the obtained NiCoBDC NRBs were redispersed in ethanol.

***Synthesis of CoTCPP NRBs*.** The pre-prepared Co(OH)_2_ nanoplates (0.01 mmol) and TCPP (0.01 mmol) were added in a mixed solution of 3 mL DMF and 1 mL deionized water in a 20 mL capped vial under stirring. After that, the vial was heated to 50 ℃ and then kept for 12 h. The resulting brownish red CoTCPP NRBs were washed twice with DMF and ethanol, respectively. Finally, the obtained CoTCPP NRBs were redispersed in ethanol.

***Synthesis of MIL-53(Al) NRBs*.** The pre-prepared AlO(OH) nanosheets (0.05 mmol) and BDC (0.05 mmol) were added in a solution of 10 mL deionized water during magnetic stirring. Then the solution was sealed in a 30 mL Teflon autoclave and heated at 160 °C for 12 h. The resulting white NRBs were washed twice with DMF and ethanol, respectively. Finally, the obtained MIL-53(Al) NRBs were redispersed in ethanol.

**Synthesis of bulk CoBDC MOF crystals.**

Bulk CoBDC was prepared according to previous report [4]. BDC (0.33 mmol) was dissolved in 16 mL DMF and 2 mL deionized water in a 50 mL beaker under stirring. Subsequently, CoCl_2_·6H_2_O (0.33 mmol) was added. The mixed solution was transferred into a 50 mL Teflon autoclave at 140 ℃ for 48 h under airtight conditions. The resulting red-brown bulk CoBDC crystals were washed twice with DMF and ethanol, respectively.

**Fluorescent DNA assays.**

In a typical hybridization experiment, 10.0 µL of probe P (2.0 µM) was hybridized with 10.0 µL of the target DNA T with different concentration (0-2.0 μM) in 1940.0 µL of phosphate buffer (1.0 ×10^-3^ M, pH 7.4) for 10 min at room temperature. Then, 40.0 µL of CoBDC NRBs solution (1.51 mg mL^−1^) was added into the aforementioned mixture. After 5 min incubation, fluorescence measurements were performed to monitor the hybridization process with the final concentration of T (0-10.0 nM). The excitation and emission wavelengths were fixed at 494 and 516 nm, respectively.

For the comparison study, 10.0 µL of probe P (2.0 µM) was hybridized with 10.0 µL of target T (2.0 μM) in 1940.0 µL of phosphate buffer (1.0 ×10^-3^ M, pH 7.4) for 10 min at room temperature. Then 40.0 μL of bulk CoBDC (1.51 mg mL^−1^) or CoBDC NRBs (1.51 mg mL^−1^) solution was added into the aforementioned mixture. After incubation for 5 min, fluorescence measurements were performed to monitor the hybridization process with the final concentration of target T (10.0 nM). The excitation and emission wavelengths were 494 and 516 nm, respectively.

To study the selectivity of CoBDC NRB-based sensor, 10.0 µL of probe P (2.0 µM) was hybridized with 10.0 µL of target T (2.0 µM), or 10.0 μL of single-base mismatch DNA (SM, 2.0 μM), or 10.0 μL of random DNA (R, 2.0 μM), respectively, in 1940.0 μL phosphate buffer (1.0 ×10^-3^ M, pH 7.4) solution for 10 min at room temperature. Then 40.0 μL of CoBDC NRBs solution (1.51 mg mL^−1^) was added into the aforementioned mixture. After incubation for 5 min, fluorescence measurements were performed to monitor the hybridization process with the final concentration of target T (10.0 nM), SM (10.0 nM), and R (10.0 nM), respectively. The excitation and emission wavelengths were 494 and 516 nm, respectively.

To test the stability of CoBDC NRBs during the DNA detection, after 10.0 µL of probe P (2.0 µM) was hybridized with 10.0 µL of target T (2.0 µM) in 1940.0 μL phosphate buffer (1.0 ×10^-3^ M, pH 7.4) solution for 10 min at room temperature, 40.0 μL of CoBDC NRBs solution (1.51 mg mL^−1^) was added and then incubated for 5 min. The mixture, also used for the selectivity test, was centrifuged at 8,000 rpm for 2 min. The obtained precipitate was washed with ethanol twice and then dried on glass for XRD measurement. The resulted supernatant fluid was used for UV-Vis absorbance analysis.

**Characterization.** Prior to the transmission electron microscopy (TEM), scanning electron microscopy (SEM) and atomic force microscopy (AFM) characterization, the ethanolic suspension of MOF NRBs was dropped onto the holey carbon-coated carbon support copper grids, Si/SiO_2_, and cleaned Si/SiO_2_ substrate, respectively. For X-ray diffraction (XRD) characterization, the ethanolic suspension of MOF NRBs was dropped onto a clean glass and dried naturally. TEM was performed on a Tecnai G^2^ 20 S-TWIN. The high-resolution TEM was operated at an acceleration voltage of 100 kV on a HT7700 Exalens. SEM images were obtained using a field emission scanning electron microscope (Zeiss SUPRA 55). AFM measurements were conducted using a Bruker multimode-8 scanning probe microscope in tapping mode in air. Powder XRD was performed on a Shimadzu XRD-6000 diffractometer with Cu Kα radiation (λ=1.5418 Å). The N_2_ adsorption-desorption isotherms and surface area were determined using an ASAP-2020 surface area analyzer at 77 K and pressure up to 1 bar. The samples were degassed at 453 K for 16 h prior to analysis. The Brunauer Emmett Teller (BET) method was used to calculate the specific surface areas. For the thermogravimetric analysis (TGA), the bulk CoBDC and CoBDC NRBs were heated at a heating rate of 10 °C/min from 10 °C to 700 °C on a Netzsch STA 449F3 instrument under a flow of dry Nitrogen with a volumetric flow rate of 100 mL/min. Fluorescence spectra were collected with a Shimadzu fluorophotometer (RF-5301PC).


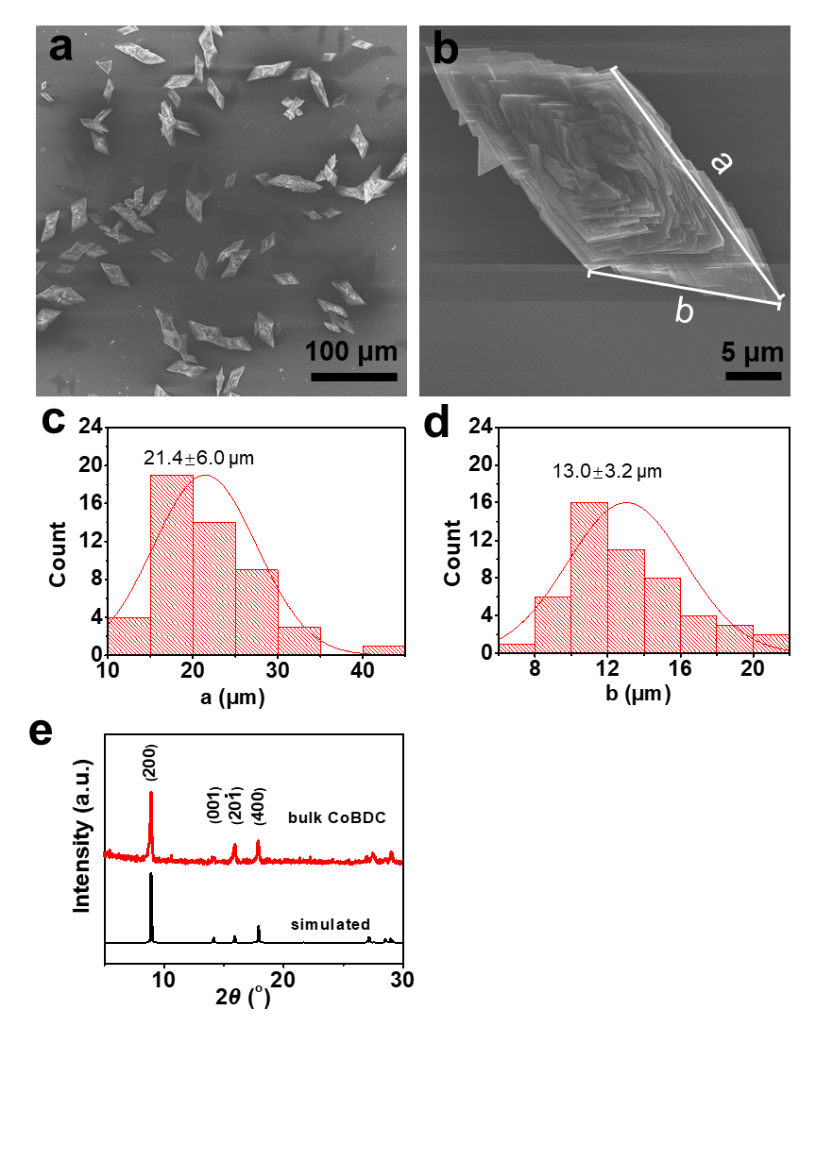


**Figure S1.** Characterization of bulk CoBDC crystals synthesized via a conventional solvothermal method. (a) Low- and (b) high-magnification SEM images of bulk CoBDC crystals. (c,d) Statistical analysis of the edge lengths of bulk CoBDC crystals measured in the SEM images. (e) XRD pattern of bulk CoBDC crystals.

**
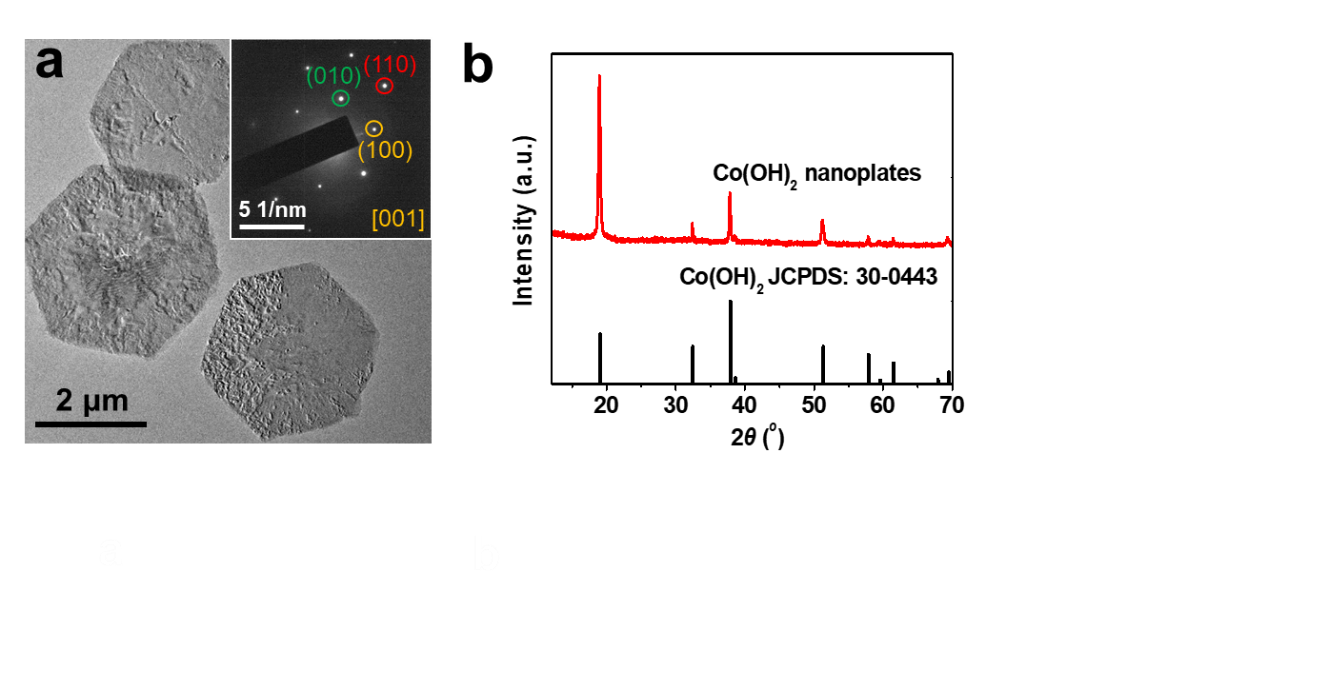
**

**Figure S2.** (a) TEM image of Co(OH)_2_ nanoplates. Inset: the corresponding SAED pattern. (b) XRD pattern of Co(OH)_2_ nanoplates.


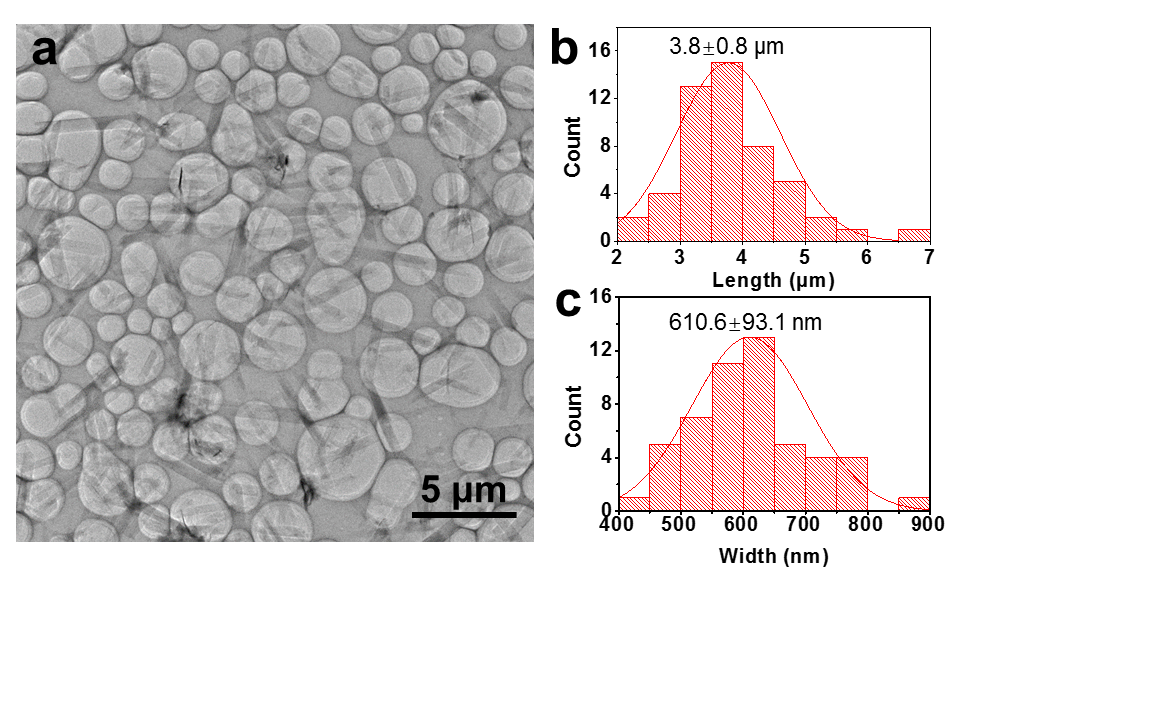


**Figure S3.** (a) Low-magnification TEM image of CoBDC NRBs. (b,c) Statistical analysis of the length (b) and width (c) of CoBDC NRBs measured in the TEM image.


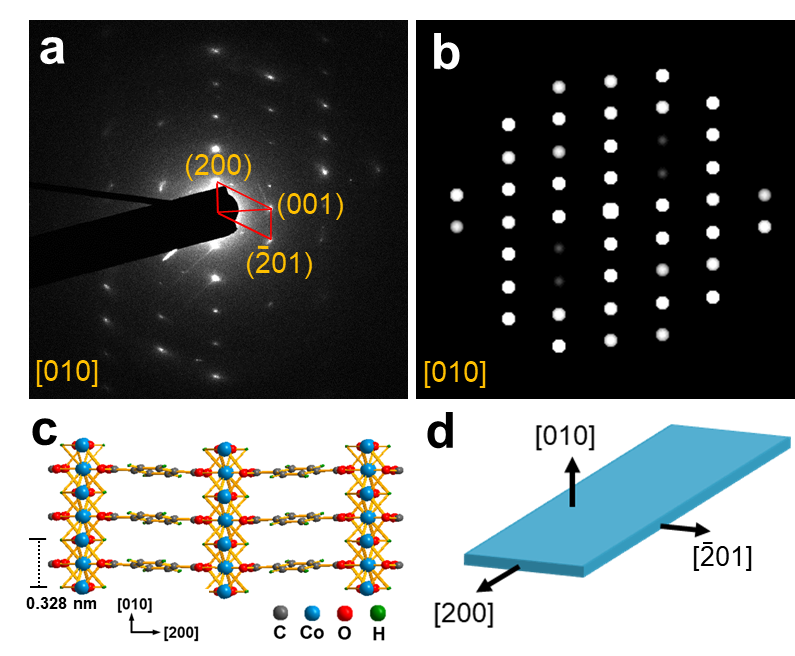


**Figure S4.** (a) SAED pattern and (b) a simulated SAED pattern of CoBDC NRB of the (010) plane. (c) Schematic illustration of the ($\bar{2}$01) lattice plane of a CoBDC NRB. (d) Simple structural model of a CoBDC NRB.


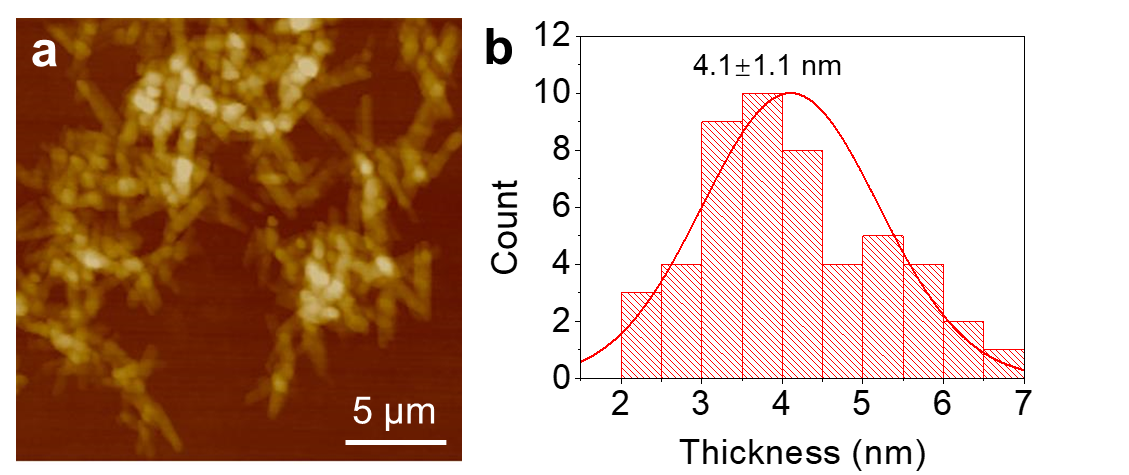


**Figure S5.** (a) AFM image of CoBDC NRBs. (b) Statistical analysis of the thickness of CoBDC NRBs measured in the AFM image.


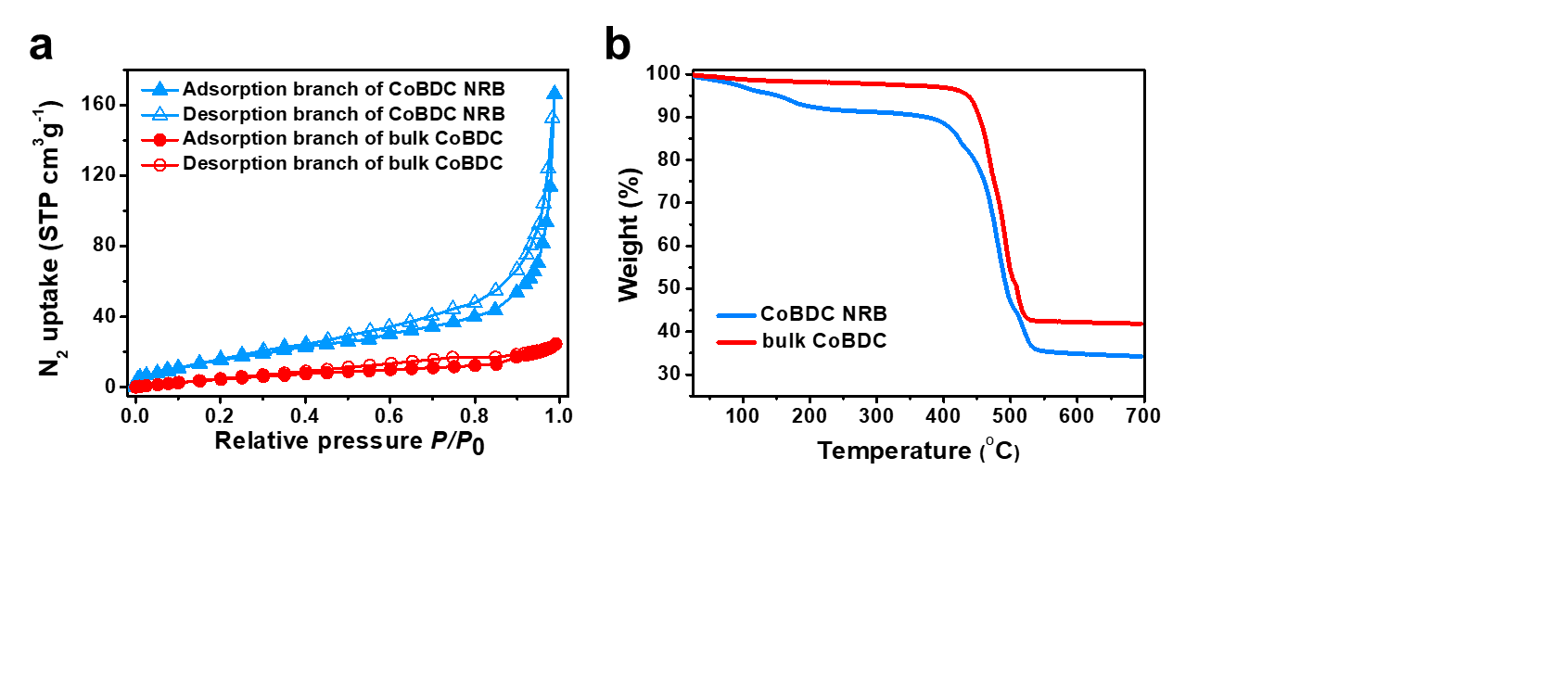


**Figure S6.** (a) N_2_ adsorption-desorption isotherms (77 K) and (b) TGA curves of CoBDC NRBs and bulk CoBDC crystals.


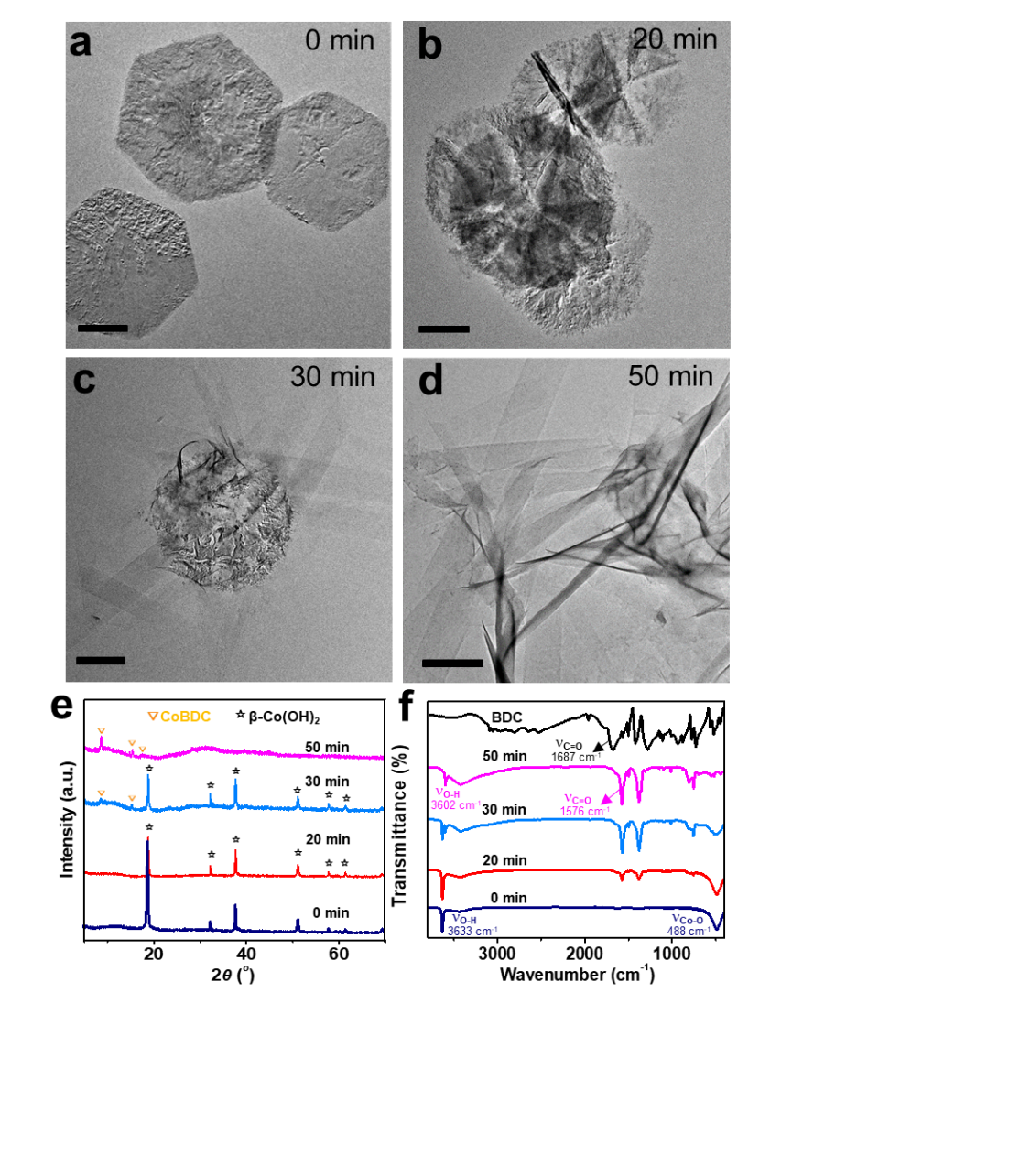


**Figure S7.** (a-d) TEM images of the products collected at the reaction time of 0 (a), 20 (b), 30 (c), and 50 (d) min, respectively. The scale bars are 1 μm. (e) XRD patterns and (f) FTIR spectra of the products collected at different reaction time of 0, 20, 30, and 50 min.

The conversion process from Co(OH)_2_ nanoplates to CoBDC NRBs was confirmed by XRD and FTIR measurements (Figure S7e, f). The XRD peaks belong to CoBDC NRBs appeared at 30 min, while those ascribed to Co(OH)_2_ nanoplates vanished at 50 min, resulting in a pure phase of CoBDC NRBs (Figure S7e). In the FTIR spectrum of Co(OH)_2_ nanoplates (Figure S7f), two peaks located at 3633 and 488 cm^−1^ are assigned to the vibrations of ν_O-H_ and ν_Co-O_ in Co(OH)_2_, respectively. After reaction with BDC for 50 min, the peaks belonging to Co(OH)_2_ disappeared and two new peaks belonging to BDC ligand appeared (ν_C=O_ at 1576 cm^−1^ and ν_C=C_ at 1383 cm^−1^). Note that the peak of ν_C=O_ in BDC ligand was slightly shifted from 1687 to 1576 cm^−1^ due to the coordination of metal ions with the ligands after the formation of CoBDC NRBs.


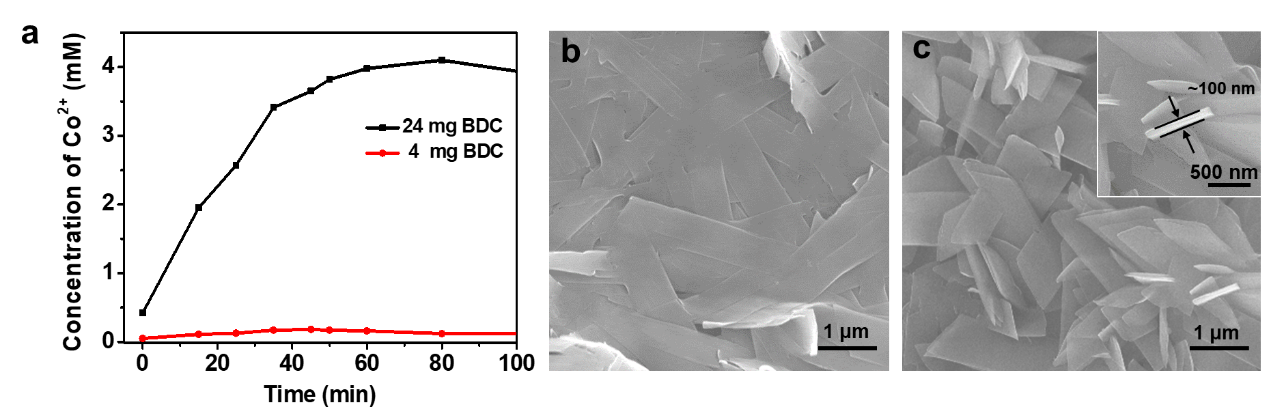


**Figure S8**. (a) Time-dependent concentration changes of Co^2+^ in the reaction solutions by using 4 mg and 24 mg BDC as ligands. (b, c) SEM images of the products obtained by using 4 mg (b) and 24 mg (c) BDC as ligands.

In a control experiment, when the excess ligand (24 mg) was used to promote the dissolution rate of Co^2+^, only irregular thick crystals were obtained due to the high concentration of Co^2+^ in the solvent (Figure S8c).


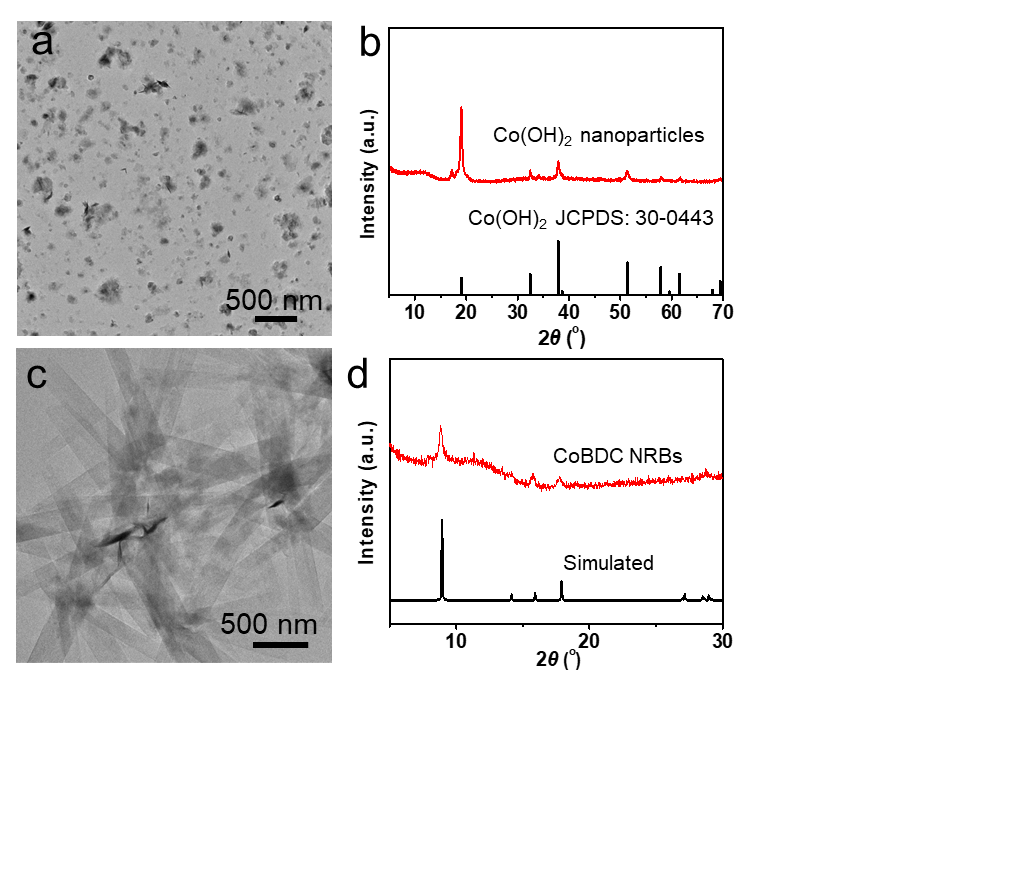


**Figure S9**. (a) TEM image and (b) XRD pattern of irregular Co(OH)_2_ nanoparticles. (c) TEM image and (d) XRD pattern of CoBDC NRBs synthesized by using the irregular Co(OH)_2_ nanoparticles as the precursors and BDC as the ligands.

When the irregular Co(OH)_2_ nanoparticles (Figure S9a, b) were used as the precursors, the CoBDC NRBs could also be obtained (Figure S9c, d), indicating that the morphology of the metal hydroxides might not affect the formation of MOF NRBs.


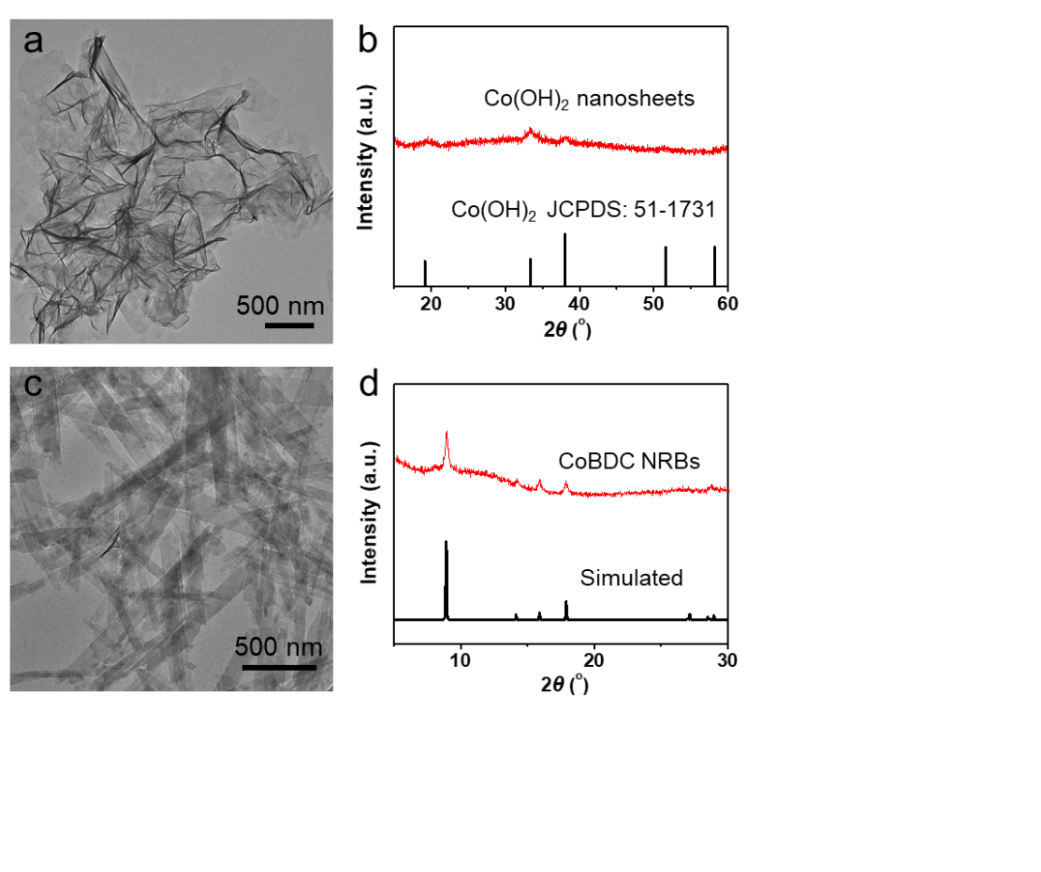


**Figure S10**. (a) TEM image and (b) XRD pattern of low crystalline Co(OH)_2_ nanosheets. (c) TEM image and (d) XRD pattern of CoBDC NRBs synthesized by using the low crystalline Co(OH)_2_ nanosheets as the precursors and BDC as the ligands.

When the low crystalline Co(OH)_2_ nanosheets (Figure S10a, b) were used as the precursors, the CoBDC NRBs were also obtained (Figure S10c, d), indicating that the crystallinity of the metal hydroxides might not affect the formation of MOF NRBs.


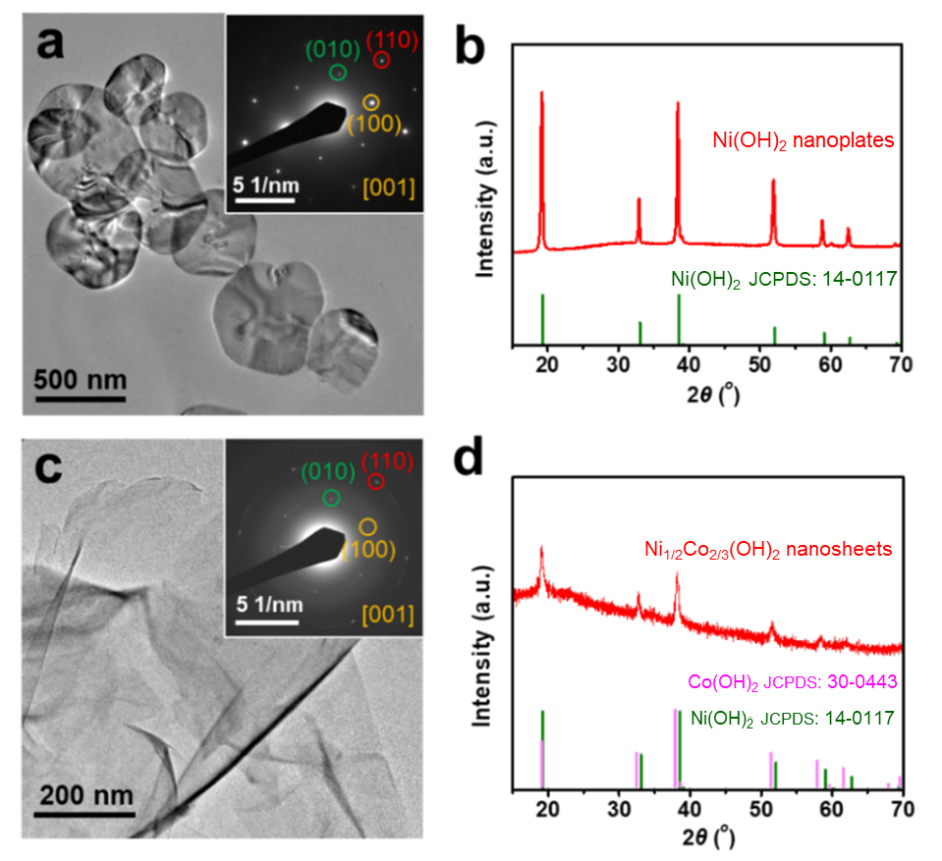


**Figure S11.** (a) TEM image and (b) XRD pattern of Ni(OH)_2_ nanoplates. Inset in (a): the corresponding SAED pattern of a Ni(OH)_2_ nanoplate. (c) TEM image and (d) XRD pattern of Ni_1/2_Co_2/3_(OH)_2_ nanosheets. Inset in (c): the corresponding SAED pattern of a Ni_1/2_Co_2/3_(OH)_2_ nanosheet.


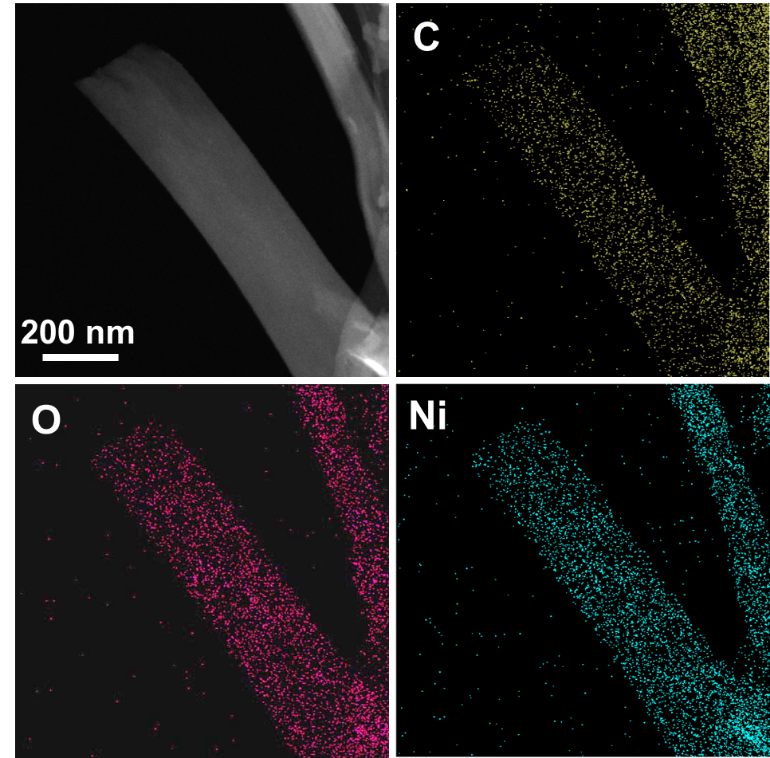


**Figure S12.** The STEM-EDS element mappings of NiBDC NRBs.


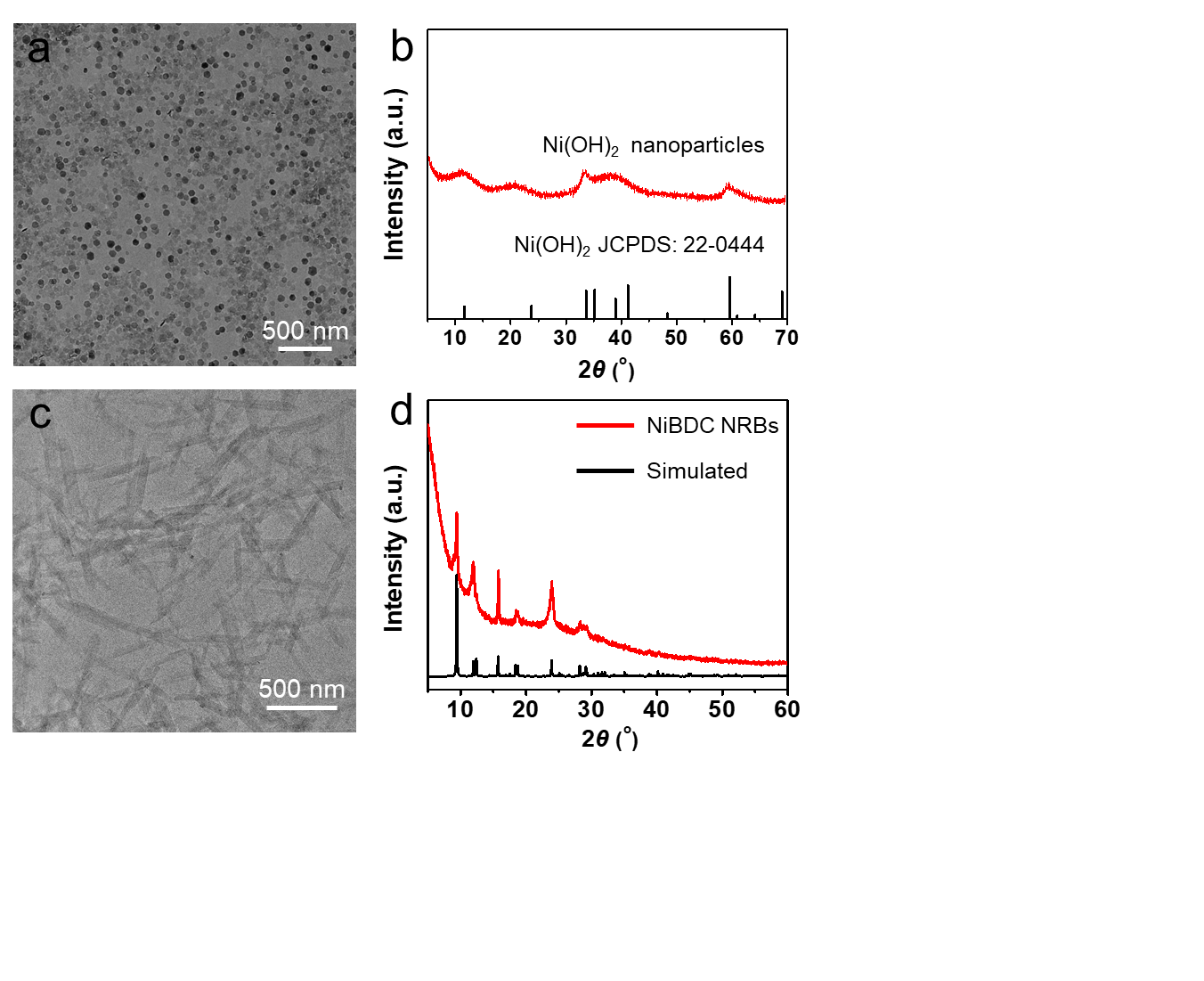


**Figure S13.** (a) TEM image and (b) XRD pattern of Ni(OH)_2_ nanoparticles. (c) TEM image and (d) XRD pattern of NiBDC NRBs synthesized by using the Ni(OH)_2_ nanoparticles as the precursors and BDC as the ligands.

When the Ni(OH)_2_ nanoparticles (Figure S13a, b) were used as the precursors, the NiBDC NRBs could also be obtained (Figure S13c, d), indicating that the morphology of the metal hydroxides might not affect the formation of MOF NRBs.


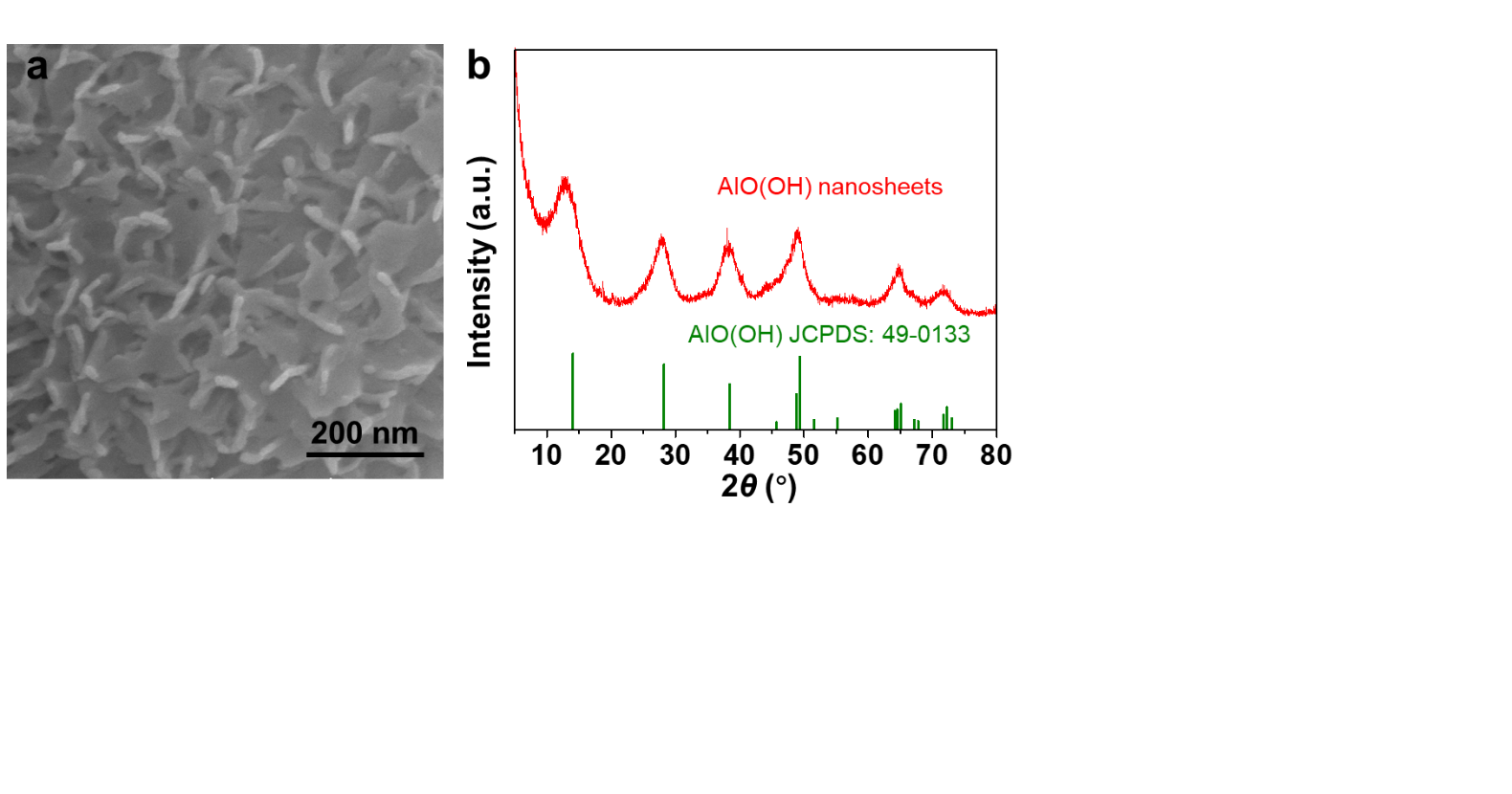


**Figure S14.** (a) SEM image and (b) XRD pattern of AlO(OH) nanosheets.


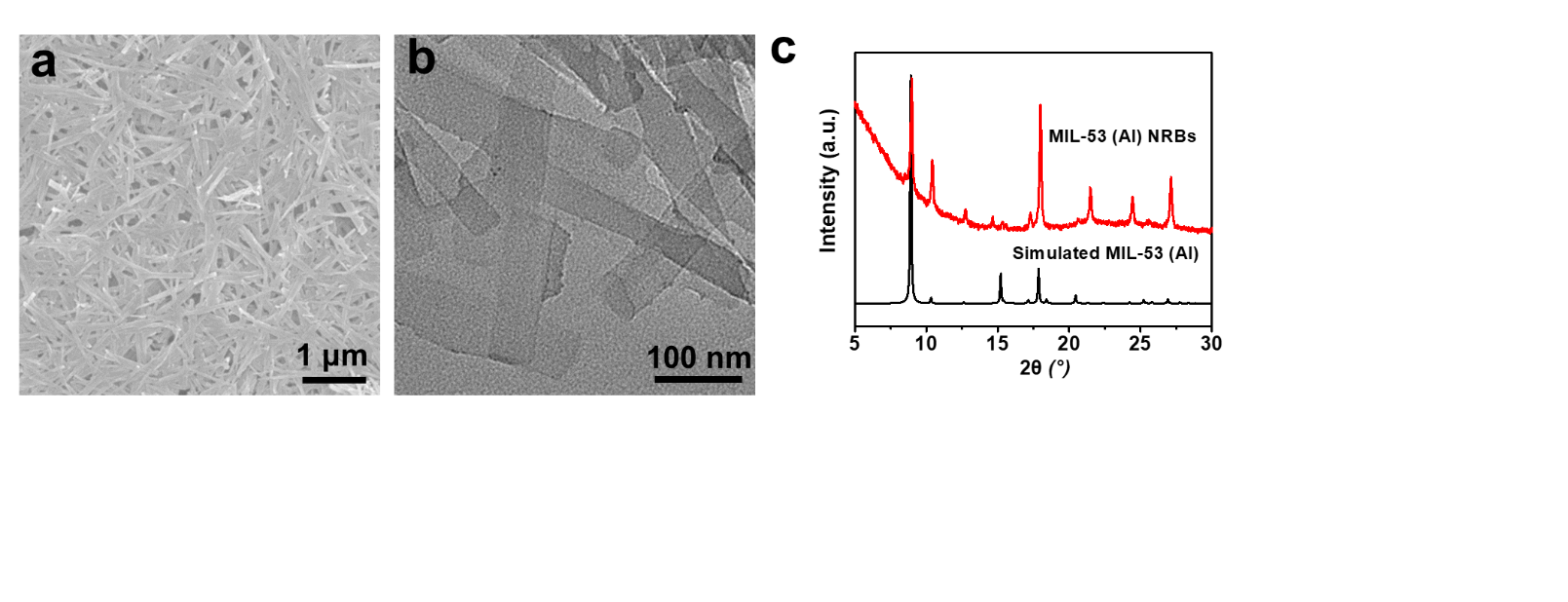


**Figure S15.** (a) SEM image, (b) TEM image and (c) XRD pattern of MIL-53(Al) NRBs synthesized by using AlO(OH) nanosheets as the precursors and BDC as the ligands.


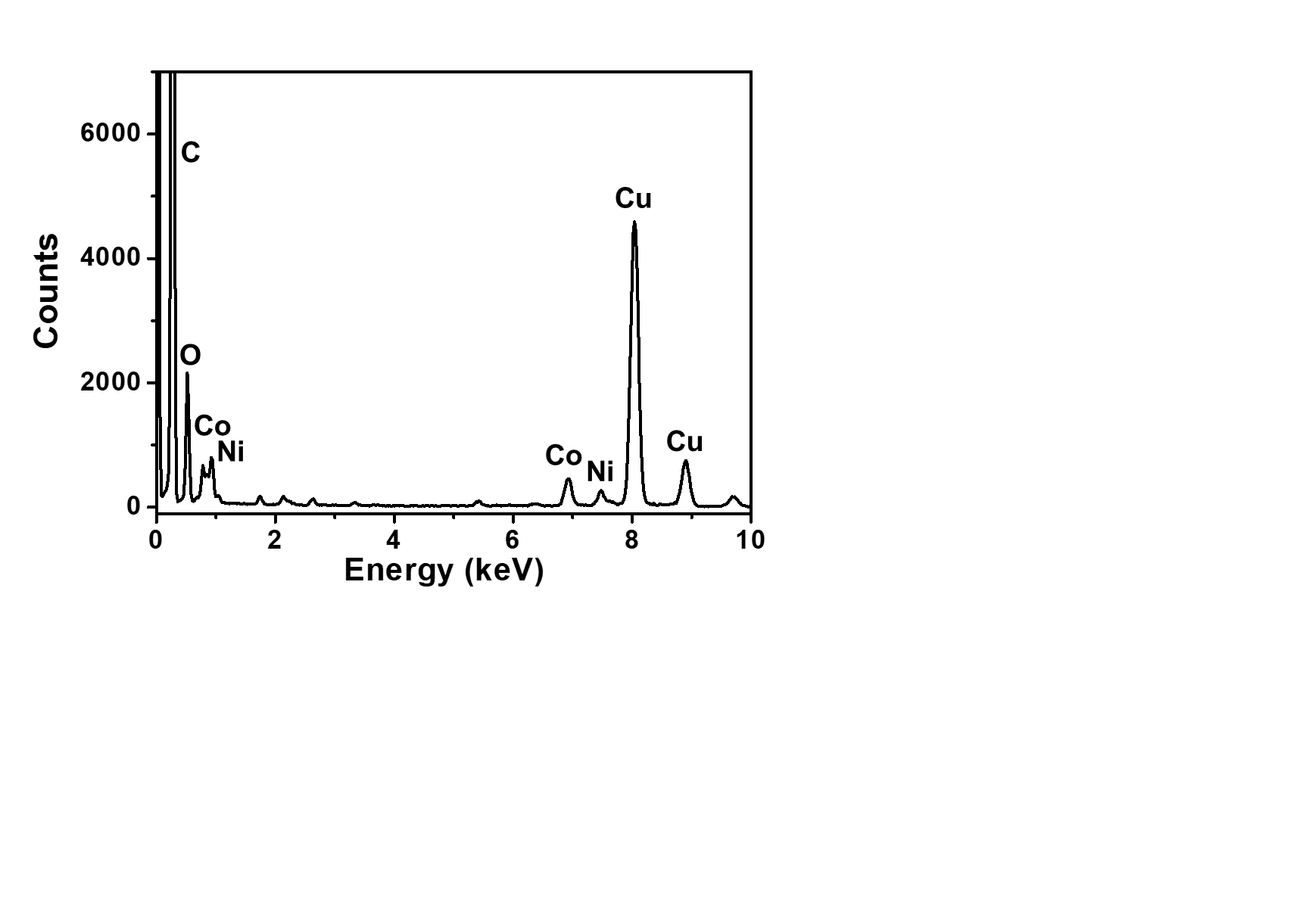


**Figure S16.** The EDS spectrum of NiCoBDC NRBs.


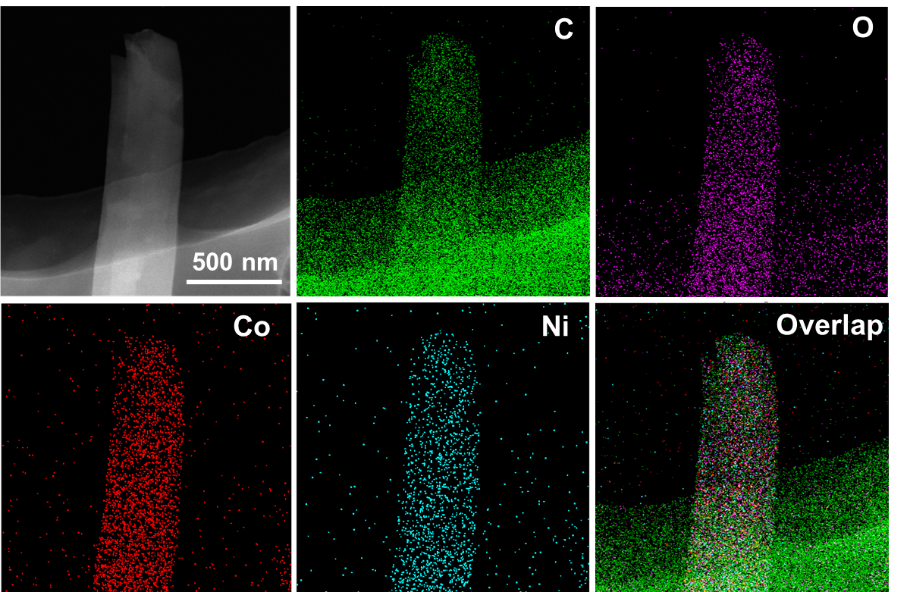


**Figure S17.** The STEM-EDS element mappings of a NiCoBDC NRB.

**
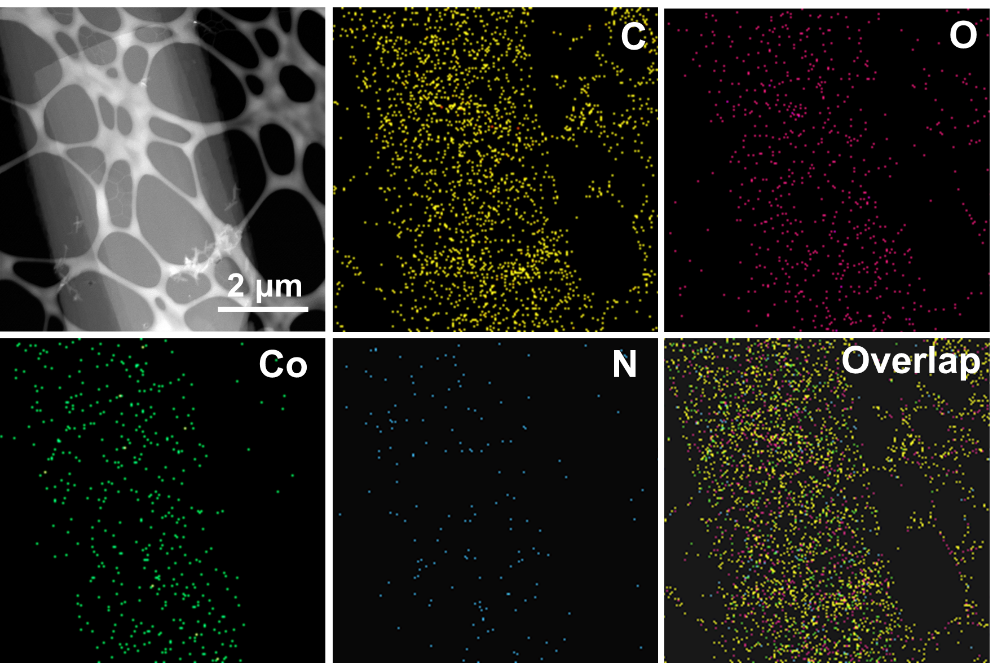
**

**Figure S18.** The STEM-EDS element mappings of CoTCPP NRBs.


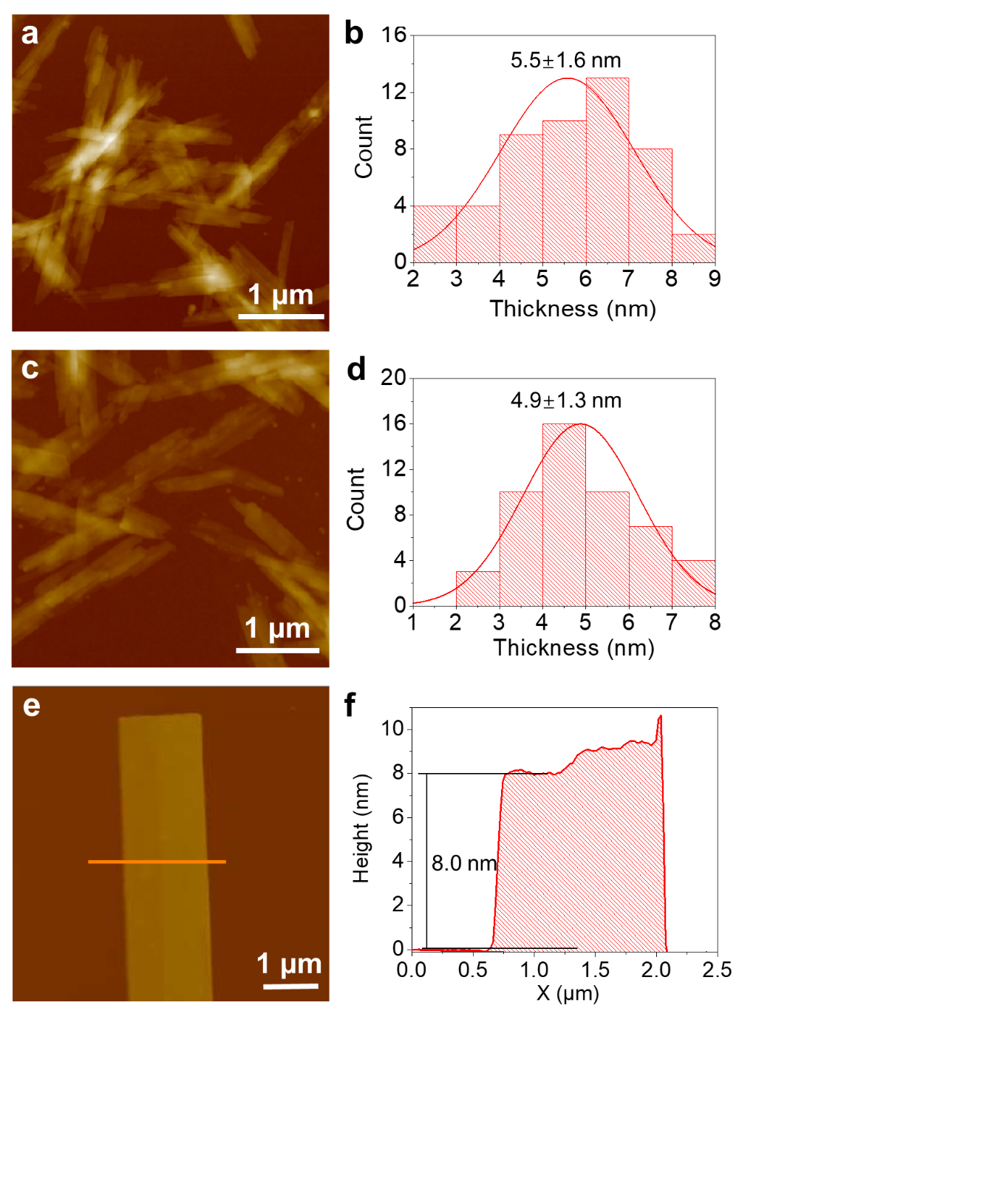


**Figure S19.** (a) AFM image of NiBDC NRBs and (b) the corresponding statistical analysis of their thickness. (c) AFM image of NiCoBDC NRBs and (d) the corresponding statistical analysis of their thickness. (e) AFM image of a CoTCPP NRB and (f) the corresponding height profile measured along the orange line in (e).

| Name | Sequence (5-3)* |
| --- | --- |
| Probe (P) | TTCTTCATCGAGAGTGTAGTCG-**FAM** |
| H1N1 (T) | CGACTACACTCTCGATGAAGAA |
| Single-base mismatch DNA (SM) | CGACTACACTCT**G**GATGAAGAA |
| Random DNA (R) | TAGCTTATCAGACAGATGTTGA |

**Table S1.** DNA sequences used for the fluorescence assay.

Abbreviation: FAM, 6-carboxyfluorescein


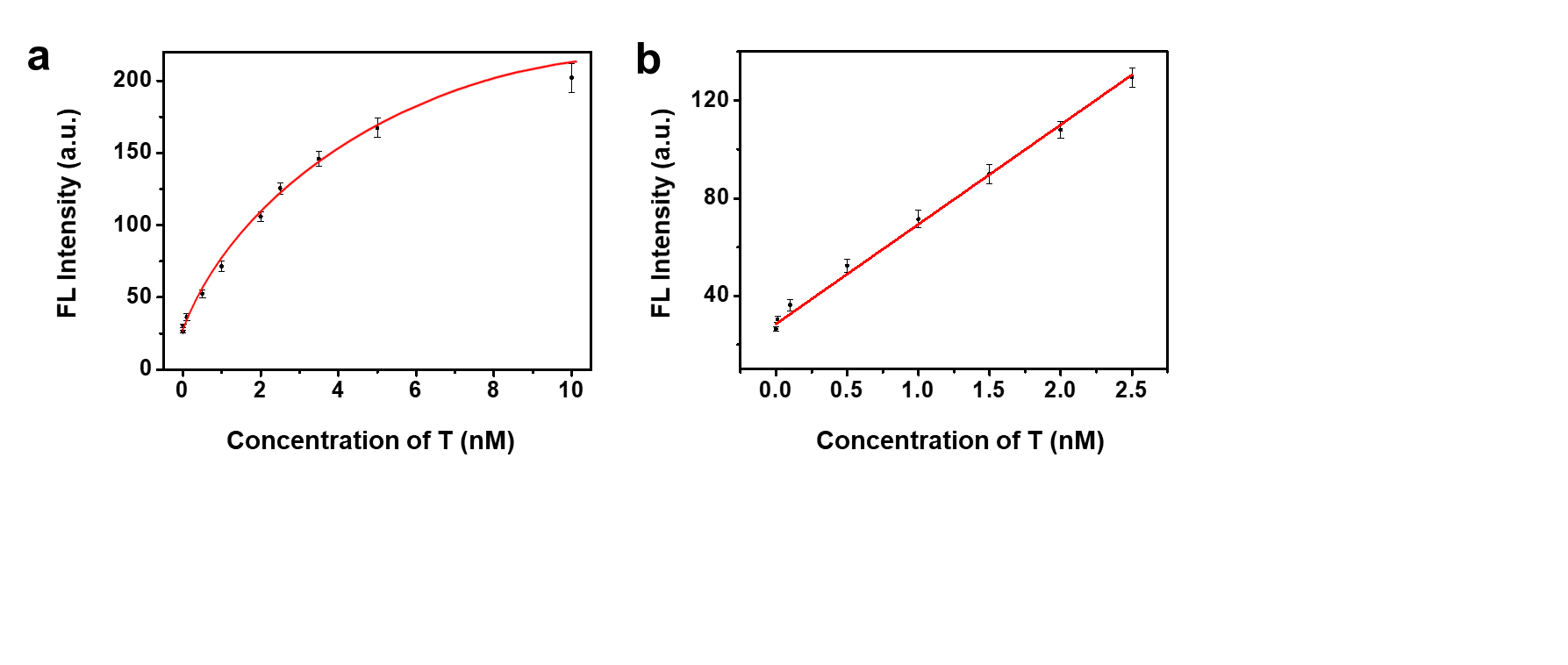


**Figure S20.** (a) Calibration curve for detection of target complementary DNA (T) using CoBDC NRB-based sensor. (b) The linear plot of fluorescence signal *vs.* concentration of target complementary DNA (T). The concentrations of P and CoBDC NRBs in the final solution are 10 nM and 30 μg mL^‒1^, respectively. Excitation and emission wavelengths are 494 and 516 nm, respectively.

**Table S2.** Comparison of fluorescent DNA sensors using different nanomaterials.

| **Nanomaterials** | **Fluorescent**  **reporter** | **Sensitivity**  **(Detection limit)** | | | **Detection**  **time** | **Ref.** |
| --- | --- | --- | --- | --- | --- | --- |
| **Other 2D material** | | | | | | |
| Graphene oxide (GO) | FAM | | 2 nM | 5 min | | [5] |
| GO | FAM, ROX, Cy5 | | 100 pM | 1 min | | [6] |
| GO | FAM, ROX | | 5 pM | 10 min | | [7] |
| g-C_3_N_4_ | FAM, ROX | | 81 pM | 30 min | | [8] |
| MoS_2_ | FAM | | 0.5 nM | 5 min | | [9] |
| TiS_2_ and TaS_2_ | FAM, Texas red | | 50 pM | 5 min | | [10] |
| COF | Texas red | | 20 pM | 15 min | | [11] |
| **MOFs and coordination polymers** | | | | | | |
| Coordination polymer nanoplates | FAM, ROX, Cy5 | | 30 pM | 70 min | | [12] |
| UiO-66-NH_2、_nanoparticles | FAM | | 10 nM | 20 min | | [13] |
| Cu(H_2_dtoa)  nanoparticles | FAM | | 3 nM | 4 h | | [14] |
| MIL-101  nanoparticles | SYBR Green I | | 73 pM | 10 min | | [15] |
| 2D Cu-TCPP  nanosheets | Texas red, TET | | 20 pM | 5 min | | [16] |
| CoBDC NRBs | FAM | | 20 pM | 5 min | | This work |

Abbreviations: ROX, 6-carboxy-x-rhodamine; Cy5, cyanine 5; TET, tetrafluororescein.


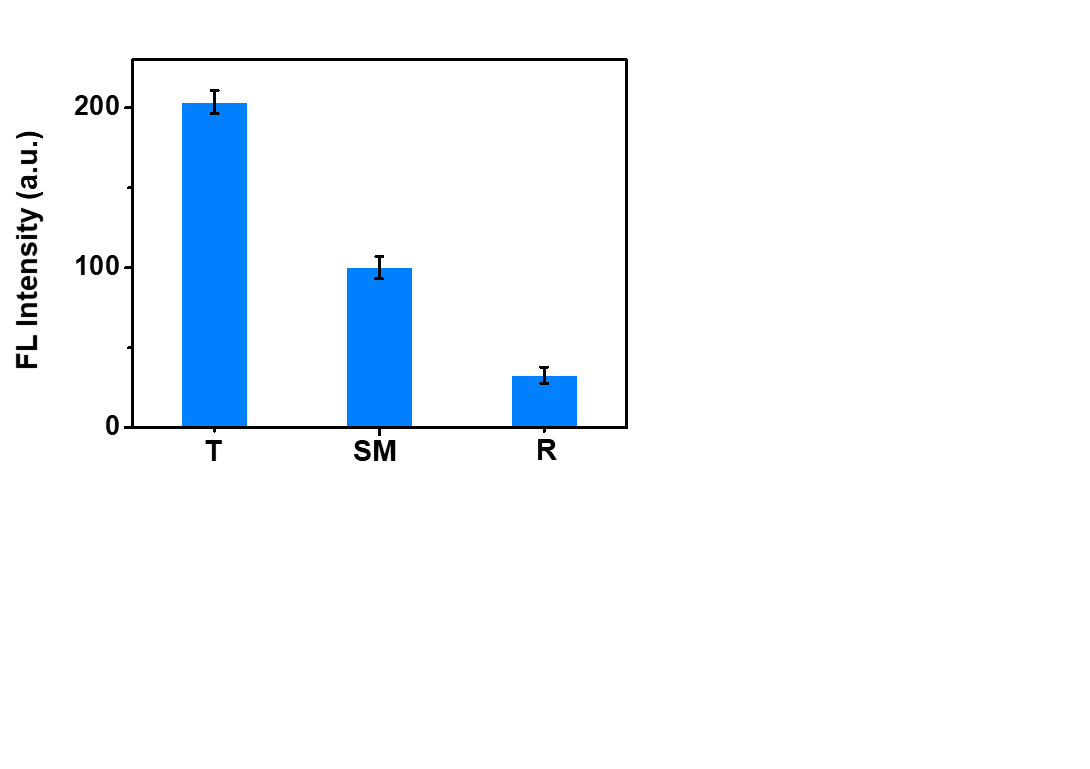


**Figure S21.** Selectivity of the CoBDC NRB-based sensor in the presence of 10 nM target DNA (T), single-base mismatched DNA (SM), or random DNA (R). Excitation and emission wavelengths are 494 and 516 nm, respectively.


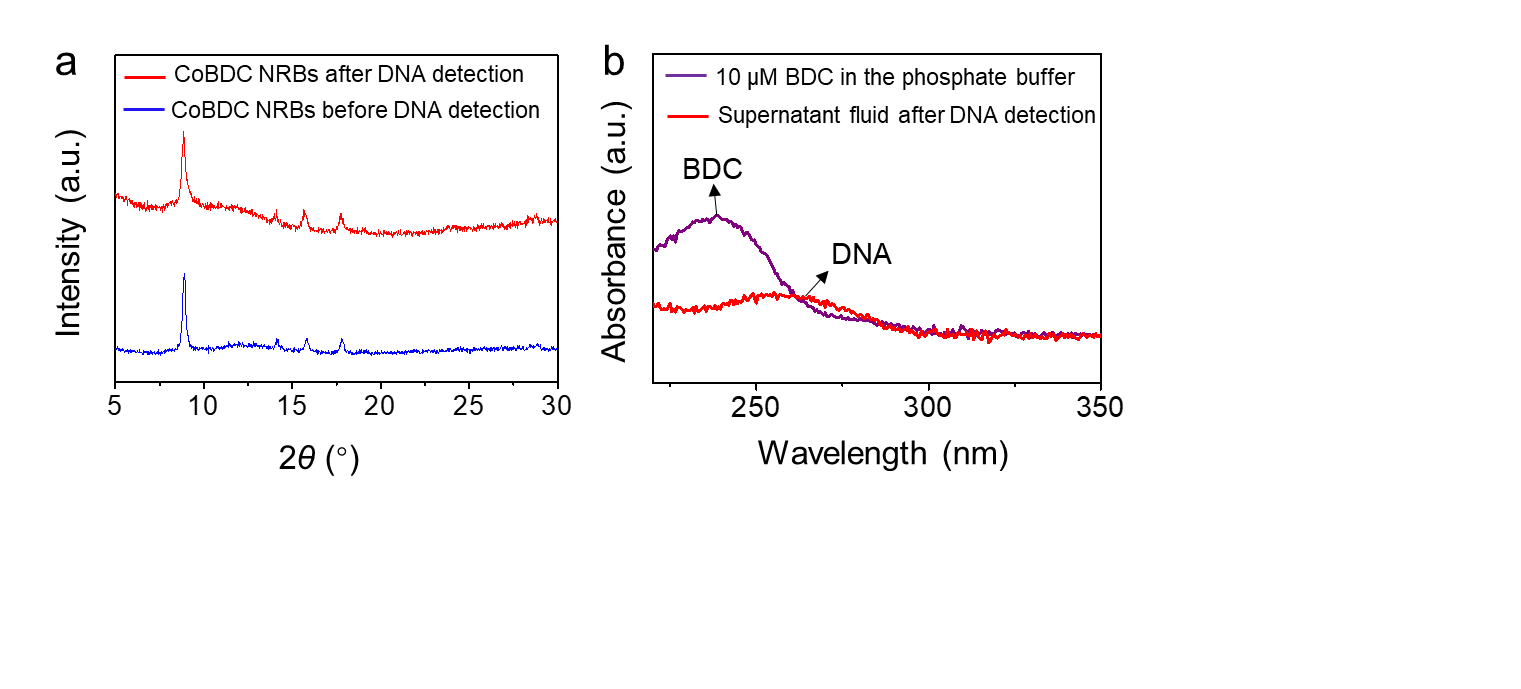


**Figure S22.** (a) XRD patterns of CoBDC NRBs before and after DNA detection. (b) UV-Vis absorbance spectra of the supernatant fluid after DNA detection using CoBDC NRBs (red line) and the 10 µM BDC in the phosphate buffer (purple line).

As shown in Figure S22a, no obvious difference was observed in the XRD patterns of the freshly prepared and used CoBDC NRBs, indicating the structure stability of CoBDC NRBs in the DNA detection, which was further confirmed by the UV-Vis analysis. As shown in Figure S22b, after the DNA detection, no absorption peak of ligands was observed in the supernatant fluid (red line), indicating no ligand leaching during the test.

**Supporting References**

1. Liu Z, Ma R, Osada M, et al., Selective and controlled synthesis of α- and β-cobalt hydroxides in highly developed hexagonal platelets. *J Am Chem Soc* 2005; **127**: 13869-74.

2. Liang Z, Zhu Y; Hu X, β-nickel hydroxide nanosheets and their thermal decomposition to nickel oxide nanosheets. *J Phys Chem B* 2004; **108**: 3488-91.

3. Song F; Hu X, Exfoliation of layered double hydroxides for enhanced oxygen evolution catalysis. *Nat Commun* 2014; **5**: 4477.

4. Zhao S, Wang Y, Dong J, et al., Ultrathin metal-organic framework nanosheets for electrocatalytic oxygen evolution. *Nat Energy* 2016; **1**: 16184.

5. Lu C, Yang H, Zhu C*, et al.*, A graphene platform for sensing biomolecules. *Angew Chem Int Ed* 2009; **48**: 4785-7.

6. He S, Song B, Li D*, et al.*, A graphene nanoprobe for rapid, sensitive, and multicolor fluorescent DNA analysis. *Adv Funct Mater* 2010; **20**: 453-9.

7. Liu X, Aizen R, Freeman R*, et al.*, Multiplexed aptasensors and amplified DNA sensors using functionalized graphene oxide: Application for logic gate operations. *ACS Nano* 2012; **6**: 3553-63.

8. Wang Q, Wang W, Lei J*, et al.*, Fluorescence quenching of carbon nitride nanosheet through its interaction with DNA for versatile fluorescence sensing. *Anal Chem* 2013; **85**: 12182-8.

9. Zhu C, Zeng Z, Li H*, et al.*, Single-layer MoS_2_-based nanoprobes for homogeneous detection of biomolecules. *J Am Chem Soc* 2013; **135**: 5998-6001.

10. Zhang Y, Zheng B, Zhu C*, et al.*, Single‐layer transition metal dichalcogenide nanosheet‐based nanosensors for rapid, sensitive, and multiplexed detection of DNA. *Adv Mater* 2015; **27**: 935-9.

11. Peng Y, Huang Y, Zhu Y*, et al.*, Ultrathin two-dimensional covalent organic framework nanosheets: Preparation and application in highly sensitive and selective DNA detection. *J Am Chem Soc* 2017; **139**: 8698-704.

12. Zhang Y, Luo Y, Tian J*, et al.*, Rectangular coordination polymer nanoplates: Large-scale, rapid synthesis and their application as a fluorescent sensing platform for DNA detection. *PLoS One* 2012; **7**: e30426.

13. Zhang H, Zhang J, Huang G*, et al.*, An amine-functionalized metal-organic framework as a sensing platform for DNA detection. *Chem Commun* 2014; **50**: 12069-72.

14. Zhu X, Zheng H, Wei X*, et al.*, Metal-organic framework (MOF): A novel sensing platform for biomolecules. *Chem Commun* 2013; **49**: 1276-8.

15. Fang J M, Leng F, Zhao X J*, et al.*, Metal-organic framework MIL-101 as a low background signal platform for label-free DNA detection. *Analyst* 2014; **139**: 801-6.

16. Zhao M, Wang Y, Ma Q*, et al.*, Ultrathin 2D metal-organic framework nanosheets. *Adv Mater* 2015; **27**: 7372-8.
